# Oral Bisphosphonates Are Associated With Increased Risk of Severe Acute Kidney Injury in Elderly Patients With Complex Health Needs: A Self‐Controlled Case Series in the United Kingdom

**DOI:** 10.1002/jbmr.4573

**Published:** 2022-06-08

**Authors:** Tetsuro Oda, Annika M. Jödicke, Danielle E. Robinson, Antonella Delmestri, Ruth H. Keogh, Daniel Prieto‐Alhambra

**Affiliations:** ^1^ Department of Medical Statistics London School of Hygiene and Tropical Medicine London UK; ^2^ Pharmaco‐ and Device Epidemiology, Centre for Statistics in Medicine, Nuffield Department of Orthopaedics, Rheumatology and Musculoskeletal Sciences University of Oxford Oxford UK

**Keywords:** AGING, GENERAL POPULATION STUDIES, STATISTICAL METHODS, ANTIRESORPTIVES

## Abstract

Although oral bisphosphonates (BP) are commonly used, there is conflicting evidence for their safety in the elderly. Safety concerns might trump BP use in older patients with complex health needs. Our study evaluated the safety of BP, focusing on severe acute kidney injury (AKI), gastrointestinal ulcer (GI ulcer), osteonecrosis of the jaw (ONJ), and femur fractures. We used UK primary care data (Clinical Practice Research Datalink [CPRD GOLD]), linked to hospital (Hospital Episode Statistics [HES] inpatient) and ONS mortality data. We included all patients aged >65 with complex health needs and no BP use in the year before study start (January 1, 2010). Complex health needs were defined in three cohorts: an electronic frailty index score ≥3 (frailty cohort), one or more unplanned hospitalization/s (hospitalization cohort); and prescription of ≥10 different medicines in 2009 (polypharmacy cohort). Incidence rates were calculated for all outcomes. Subsequently, all individuals who experienced AKI or GI ulcer anytime during follow‐up were included for Self‐Controlled Case Series (SCCS) analyses. Incidence rate ratios (IRRs) were estimated separately for AKI and GI ulcer, comparing event rates between BP‐exposed and unexposed time windows. No SCCS were conducted for ONJ and femur fractures. We identified 94,364 individuals in the frailty cohort, as well as 78,184 and 95,621 persons in the hospitalization and polypharmacy cohorts. Of those, 3023, 1950, and 2992 individuals experienced AKI and 1403, 1019, and 1453 had GI ulcer/s during follow‐up, respectively. Age‐adjusted SCCS models found evidence of increased risk of AKI associated with BP use (frailty cohort: IRR 1.65; 95% confidence interval [CI], 1.25–2.19), but no association with GI ulcers (frailty cohort: IRR 1.24; 95% CI, 0.86–1.78). Similar results were obtained for the hospitalization and polypharmacy cohorts. Our study found a 50% to 65% increased risk of AKI associated with BP use in elderly patients with complex health needs. Future studies should further investigate the risk–benefit of BP use in these patients. © 2022 The Authors. *Journal of Bone and Mineral Research* published by Wiley Periodicals LLC on behalf of American Society for Bone and Mineral Research (ASBMR).

## Introduction

Oral bisphosphonates (BPs) are antiresorptive agents widely used as first‐line treatments for prevention of fragility fractures, with several trials highlighted their efficacy in postmenopausal osteoporotic women.^(^ ^1^ ^)^ Although fracture risk is increased in the elderly and preventative treatment is important for those individuals at high fracture risk, the expected benefits from preventative treatments might be reduced in multimorbid people with a limited life expectancy, as highlighted in the National Institute for Health and Care Excellence (NICE) multimorbidity guideline.^(^ ^2^ ^)^ In addition, age‐related physiological changes such as decreased kidney function, multimorbidity, and polypharmacy make the elderly more prone to adverse drug events.

Although BPs are generally safe and well‐tolerated, they have been associated with adverse events including gastrointestinal (GI) intolerance, nephrotoxicity, and rare skeletal side effects (osteonecrosis of the jaw [ONJ] or atypical femur fracture).^(^ ^3^, ^4^, ^5^, ^6^ ^)^ GI intolerance is among the most common side effects, resulting in discontinuation of BP in up to 20% of patients.^(^ ^7^ ^)^ Adverse GI events include dysphagia, esophagitis, and severe GI disease such as upper GI ulcer, which are associated with increased bleeding risk and present a risk factor for GI cancer development. However, evidence of risk for severe GI side effects is inconclusive in the literature and high background rates of GI problems were reported in the elderly. Because reduced kidney function is common in the elderly, renal safety is of particular relevance in this patient group. Nephrotoxicity has been reported for intravenous BP in cancer patients,^(^ ^8^ ^)^ suggesting a dose‐dependent and infusion time–dependent association.^(^ ^9^ ^)^ With BP predominantly excreted via the kidneys, BPs are not recommended in patients with severe kidney disease because data on BP use in patients with renal impairment is scarce. Although no significant nephrotoxicity has been reported in larger studies for oral BPs, there remain widely held concerns in clinical practice.

Because uncertainty regarding BP safety in elderly frail patients might trump their use in this patient population, our objective was to study the association between BP use and adverse events, which were previously associated with BP, in elderly patients with complex health needs. Particularly, we assessed risk for two severe adverse event—severe acute kidney injury (AKI) and upper GI ulcer—in self‐controlled case series (SCCS), a study design particularly suited to controlling confounding when studying the frail elderly.

## Subjects and Methods

### Data source

Our study is based on data extracted from the Clinical Practice Research Datalink (CPRD GOLD), a primary care dataset containing anonymized records for >17 million current and historical patients in the UK.^(^ ^10^ ^)^ The dataset is considered representative of the UK population, with respect to age, sex, and ethnicity and comprises data on patient demographics, diagnoses, drug prescriptions, laboratory tests, and health‐related lifestyle factors, as well as secondary care events and referrals.^(^ ^11^ ^)^ We used CPRD GOLD data linked to Hospital Episode Statistics Admitted Patient Care (HES APC) inpatient data, the Office for National Statistics (ONS) mortality data and Index of Multiple Deprivation (IMD).

### Study population

**Fig. 1:**
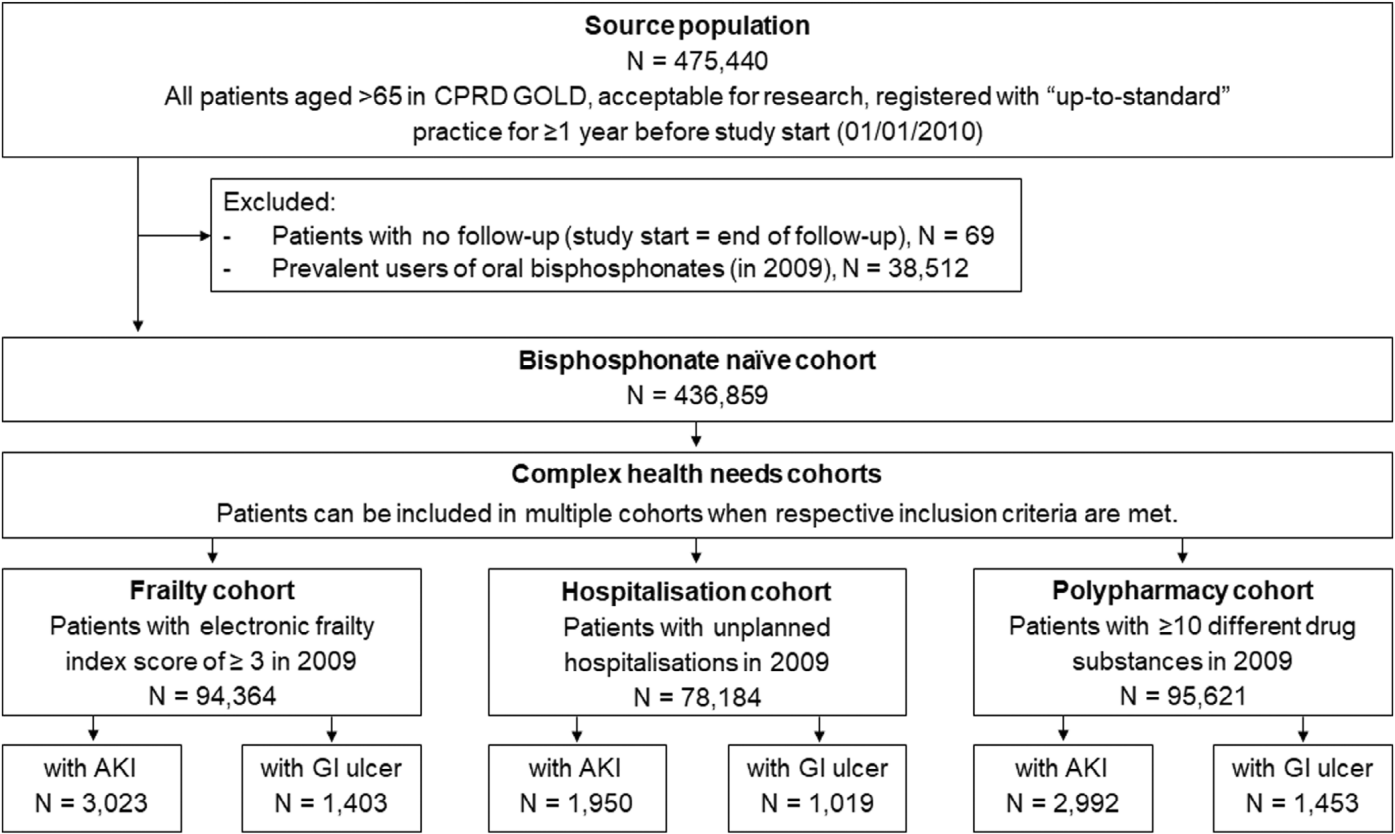
Study population flowchart. AKI = acute kidney injury; CPRD GOLD = Clinical Practice Research Datalink GOLD dataset; GI ulcer = gastrointestinal ulcer; N = number of patients.

We included all patients aged >65 years at study start (January 1, 2010) who were registered with an up‐to‐standard practice in CPRD GOLD for at least 1 year before cohort entry. Available data linkage to HES and ONS was required for all patients as well as a minimum follow‐up duration of 1 day. We subsequently excluded patients exposed to BPs in the year before study start. Among the remaining patients, we identified three cohorts of patients with complex health needs: We included patients with (i) an electronic frailty index score^(^ ^12^ ^)^ of ≥3 in the year before study start in the frailty cohort. The validated electronic frailty index has been developed by Clegg and colleagues^(^ ^12^ ^)^ based on 36 predefined deficits and is used by the National Health Service (NHS) to support routine frailty identification. Patients with (ii) unplanned hospitalizations in 2009 comprised the hospitalization cohort, and (iii) ≥10 different medications prescribed in 2009 were included in the polypharmacy cohort. A study population flowchart is provided in Fig. 1.

Patients were followed from the study start until the earliest of patient leaving practice, practice last data collection date, end of HES AP linkage coverage, death^(^ ^13^ ^)^ or date of data extraction (September 13, 2019).

### Outcomes

The outcomes of interest were AKI, upper GI ulcer, ONJ, and fractures of the subtrochanteric/distal femur or femur shaft.

AKI was defined as severe acute kidney injury leading to hospitalization. Events were identified based on International Classification of Diseases and Related Health Problems, 10th Revision (ICD‐10) codes N17 and N19, as defined in previous studies,^(^ ^14^, ^15^ ^)^ and must have been recorded as the main diagnosis leading to hospitalization in HES. A previous validation study based on ICD‐10 N17 showed that 95% of validated cases met the KDIGO definition for AKI.^(^ ^14^ ^)^

All other outcomes were identified from both CPRD based on recorded READ codes (Table S1) and main hospital diagnosis in HES (GI ulcer: ICD‐10 codes K25–28, ONJ: K10.2, subtrochanteric, shaft, or distal femoral fractures: S72.2, S72.3, S72.4). The date of each event or diagnosis was available. For all outcomes, we deemed subsequent events recorded within 30 days of an initial event to be duplicates and removed them from the dataset.

### Exposure

Oral BP prescriptions were identified using product‐specific codes in CPRD (Table S2). Exposure start was the date of the first new BP prescription for each patient in CPRD. The duration of each prescription was estimated using (i) CPRD GOLDs Therapy and Common dosages tables when acceptable number of days or quantity/daily doses were available or (ii) the most frequent duration for the prescription product in the current dataset when other data were not present. Continuous treatment episodes were constructed from the prescriptions using the method proposed by Gardarsdottir and colleagues^(^ ^16^ ^)^: episodes were based on periods of continuous BP use, defined as no refill gaps of >90 days between repeat prescriptions. A gap length of 180 days was used for sensitivity analysis. A 90‐day washout period was added to the end of each continuous treatment episode to account for noncompliance and stockpiling.

### Self‐controlled case series

The self‐controlled case series (SCCS) is an intraindividual study design, comparing event rates between exposed and unexposed patient time in patients who experienced the event of interest.^(^ ^17^, ^18^, ^19^ ^)^ The main advantage of the SCCS method is that patients serve as their own control, meaning that time‐invariant confounding is controlled for. However, confounding of the association between BP use and the outcome by time‐dependent variables remain to be addressed. Our SCCS included all patients who had a recording of AKI or GI ulcer/s during follow‐up, with patients not being required to initiate BPs during the study. Patients who are unexposed during the whole study period only contribute information to the estimation of age‐specific event rates under non‐exposure to BP. Figure S1 illustrates the study design. Exposure periods started the day after prescription date.

The SCCS method relies on strong assumptions, three of which are relevant to this study: (i) recurrent events of the same event type within the same individual should be independent from each other or rare; (ii) observation periods should be independent of occurrence of events; and (iii) the occurrence of an event should not affect the probability of subsequent exposure.^(^ ^19^ ^)^

### Statistical analysis

All analyses were conducted separately for the hospitalization, frailty, and polypharmacy cohorts. Incidence rates for all outcomes were calculated per 100,000 person‐years assuming Poisson distribution: all persons in the respective cohorts who did not have a recorded history of the respective safety outcome were included for incident rate (IR) calculation, and patient time was calculated from study start until the first event or end of follow‐up, whichever came first.

SCCS were conducted for AKI and GI ulcer but could not be undertaken for ONJ and subtrochanteric, shaft or distal femoral fractures due to small sample size and violation of SCCS model assumptions, respectively. IR ratios (IRRs) were estimated using conditional Poisson regression. These analyses were performed in R (version 4.0.3) using the SCCS package (R Foundation for Statistical Computing, Vienna, Austria; https://www.r-project.org/).^(^ ^20^ ^)^ Our main models were adjusted for age using 1‐year age bands to address time‐varying confounding. Sensitivity analyses (i) using only the first event per patient; (ii) restricting to only patients who survived during follow‐up (for AKI only); and (iii) adding a pre‐exposure washout period (AKI: 183 days, GI ulcer: 30 days) were used to test if the SCCS model assumptions hold for our study. Additional sensitivity analyses are included in Tables S3c/S4c.

## Results

The frailty cohort, hospitalization cohort, and polypharmacy cohort included 94,364, 78,184, and 95,621 individuals, who did not use BPs in the year before study start, respectively. In the following, we report results retrieved from the frailty cohort. Results derived from the hospitalization and polypharmacy cohorts are included in (Tables S1a/b, S2, S3a/b/c, S4a/b/c).

### Incidence rates

Incidence rates per 100,000 person‐years for the frailty cohort were 774.3 for AKI (95% CI, 745.8–802.7), 354.9 for GI ulcer (95% CI, 335.4–374.4), 1.6 for ONJ (95% CI, 0.3–2.9), and 129.0 for subtrochanteric, shaft, or distal femoral fractures (95% CI, 117.5–140.6) (Table S2).

### SCCS

#### Cohort characteristics

Among those included in the frailty cohort, 3023 (3.2%) individuals experienced severe AKI, accounting for 3302 AKI events recorded within 375,090 person‐years of follow‐up. Of these, 307 patients were exposed to BPs, with 171 AKI events recorded while on treatment. A total of 1403 (1.7%) patients in the frailty cohort had at least one recording for a GI ulcer event, contributing 1603 events. Among those, 152 were exposed to BP during follow‐up with 67 GI ulcer events recorded during exposed patient time. Table 1 summarizes the characteristics for patients with severe AKI and GI ulcer, among whom the SCCS analyses were conducted.

**Table 1:** Patient Demographics: Patients in Frailty Cohort With AKI or GI Ulcer, Respectively

	AKI		GI ulcer	
Characteristic	Exposed	Unexposed	Exposed	Unexposed
*n*	307	2,716	152	1,251
Age (years)	80 [75, 85]	80 [74, 85]	77 [73, 83]	78 [72, 83]
Gender (female)	213 (69)	1,217 (45)	95 (62)	563 (45%)
Follow‐up (years)	5.12 [3.61, 6.78]	4.21 [2.37, 5.96]	5.59 [4.01, 7.10]	4.53 [2.86, 6.31]
Total treatment duration (days)	491 [94, 935]	NA	478 [238, 865]	NA
Number of treatment episodes				
0	0 (0)	2,716 (100)	0 (0)	1,251 (100%)
1	250 (81)	0 (0)	121 (80)	0 (0%)
2	44 (14)	0 (0)	25 (16)	0 (0%)
≥3	13 (4.2)	0 (0)	6 (3.9)	0 (0%)
Number of respective outcome events^a^				
1	283 (92)	2,502 (92)	135 (89)	1,102 (88%)
2	20 (6.5)	184 (6.8)	14 (9.2)	127 (10%)
3	<5 (1.0)	25 (0.9)	<5 (1.3)	16 (1.3%)
≥4	<5 (0.3)	5 (0.1)	<5 (0.7)	6 (0.4%)
Index of multiple deprivation				
1 (most deprived)	79 (26)	538 (20)	36 (24)	261 (21%)
2	65 (21)	626 (23)	35 (23)	308 (25%)
3	60 (20)	594 (22)	20 (13)	254 (20%)
4	64 (21)	550 (20)	36 (24)	234 (19%)
5 (least deprived)	39 (13)	407 (15)	25 (16)	192 (15%)
Missing	0 (0)	<5 (<0.1)	0 (0)	<5 (0.2%)
Body mass index (5 years)				
Underweight	6 (2.0)	34 (1.3)	<5 (1.3)	15 (1.2%)
Normal	76 (25)	580 (21)	51 (34)	283 (23%)
Overweight	93 (30)	862 (32)	50 (33)	454 (36%)
Obese	112 (36)	982 (36)	32 (21)	387 (31%)
Missing	20 (6.5)	258 (9.5)	17 (11)	112 (9.0%)
Drinking status (5 years)				
Drinker	19 (5.9)	160 (5.9)	10 (6.6)	108 (5.6%)
Ex‐drinker	97 (32)	994 (37)	41 (27)	453 (36%)
Non‐drinker	99 (32)	821 (30)	50 (33)	365 (29%)
Missing	92 (30)	741 (27)	51 (34)	325 (26%)
Smoking status (5 years)				
Smoker	25 (8.1)	219 (8.1)	13 (8.6)	142 (8.6%)
Ex‐smoker	133 (42)	1,296 (48)	62 (41)	589 (36%)
Non‐smoker	141 (46)	1,129 (42)	74 (49)	492 (29%)
Missing	8 (2.6)	72 (2.7)	<5	28 (2.2%)
Number of different drugs (1 year)				
<5	7 (2.3)	44 (1.6)	<5 (2.6)	23 (1.8%)
5–9	78 (25)	892 (33)	48 (32)	482 (39%)
10–15	121 (39)	1,015 (37)	51 (34)	451 (36%)
>15	101 (33)	765 (28)	49 (32)	295 (24%)
Charlson Comorbidity Index (1 year)				
0	218 (71)	1,757 (65)	116 (76)	876 (70%)
1	37 (12)	342 (13)	18 (12)	149 (12%)
2	44 (14)	449 (17)	13 (8.6)	168 (13%)
≥3	8 (2.6)	168 (6.2)	5 (3.3)	58 (4.6%)
eFI (1 year)	3 [3, 5]	4 [3, 5]	3 [3, 4]	3 [3, 4]
Number of GP visits (1 year)	15 [10, 22]	14 [9, 23]	15 [9, 23]	14 [9, 21]
Fracture before BP initiation (1 year)	101 (33)	NA	68 (45)	NA
History of respective outcome^a^	18 (5.9)	170 (6.3)	11 (7.2)	121 (9.7%)
History of osteoporosis^b^	15 (4.9)	75 (2.8)	12 (7.9)	26 (2.1%)
History of chronic renal impairment^b^	165 (54)	1,657 (61)	60 (39)	529 (42%)
Death [Died]	168 (55)	1,615 (59)	69 (45)	508 (41%)

Statistics presented as median [IQR] or *n* (%). For each person, the most recent recording within 5 years prior to study start was considered for BMI, drinking status, and smoking status, or labeled “missing” of no recording was available in that timeframe. Number of different drugs, number of GP visits Charlson Comorbidity index, eFI were calculated based on the year before study start. Fracture before BP initiation (yes/no) was calculated based on the year before the first BP prescription for people with BP prescriptions.

BP = oral bisphosphonates; eFI = electronic frailty index, GP = general practitioner; NA = treatment duration and date of BP initiation not available for BP unexposed people.

^a^ AKI events for AKI cohort, GI ulcer event for GI ulcer cohort.

^b^ Any time in patient history.

#### Severe AKI

**Fig. 2:**
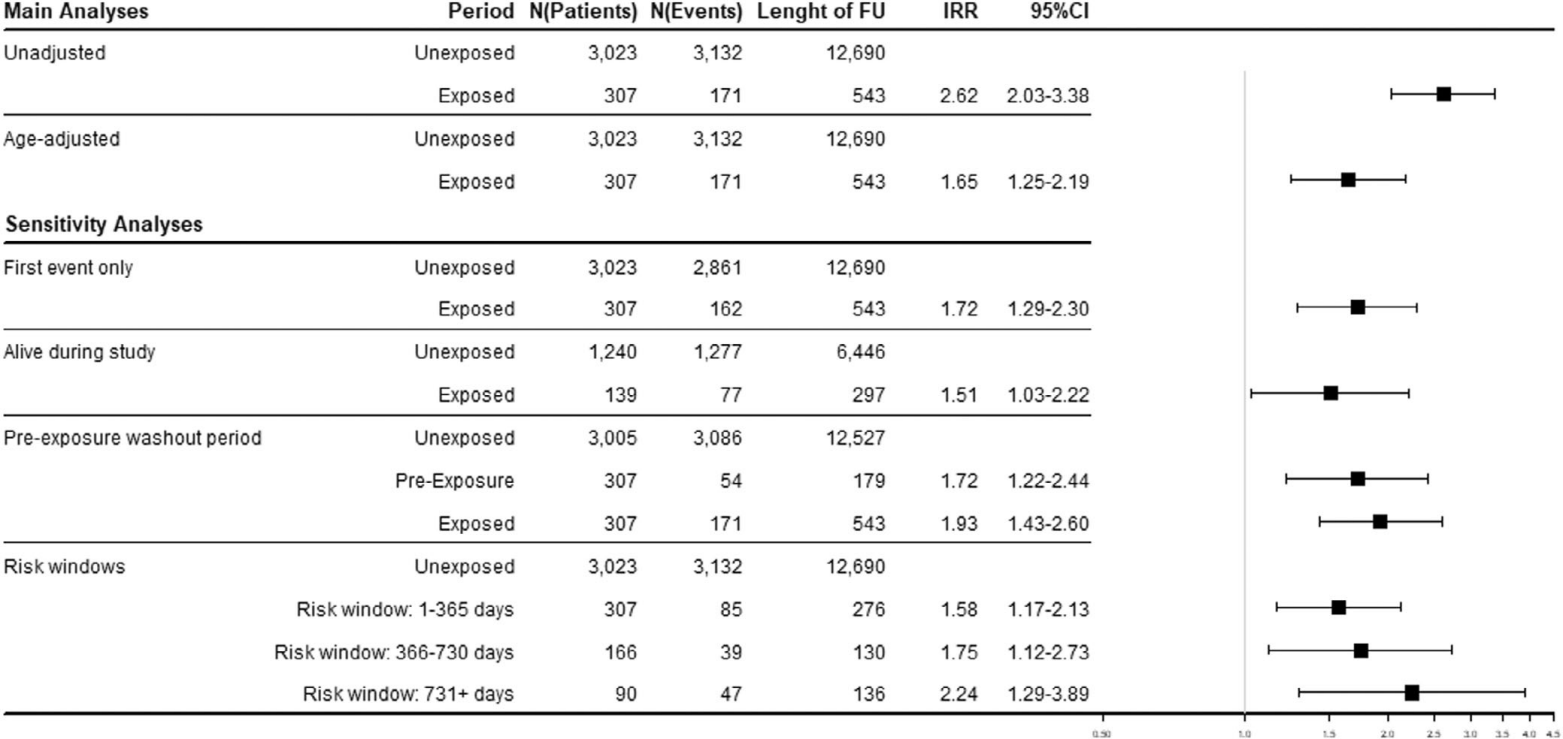
Results from SCCS analyses for severe AKI (frailty cohort). AKI = acute kidney injury; BP = oral bisphosphonates; IRR = incidence rate ratio.

The unadjusted SCCS model gave an estimated IRR of 2.62 (95% CI, 2.03–3.38) for the association between BP use and AKI in the frailty cohort. After adjustment for age, the IRR was 1.65 (95% CI, 1.25–2.19) (Fig. 2). Results obtained from the analyses conducted in the hospitalization and polypharmacy cohorts were largely comparable, with IRR 1.50 (95% CI, 1.05–2.12) and IRR 1.60 (95% CI, 1.22–2.08) for the age‐adjusted analyses, respectively (Table S3a/b).

Sensitivity analyses to test the assumptions of the SCCS model were consistent with our main results (Fig. 2). When splitting the exposure period into risk windows based on length of continuous BP treatment, no significantly increased risk was found associated with longer treatment duration. Additional sensitivity analyses show largely similar results, with only one analysis becoming nonsignificant (Table S3c).

#### GI ulcer

**Fig. 3:**
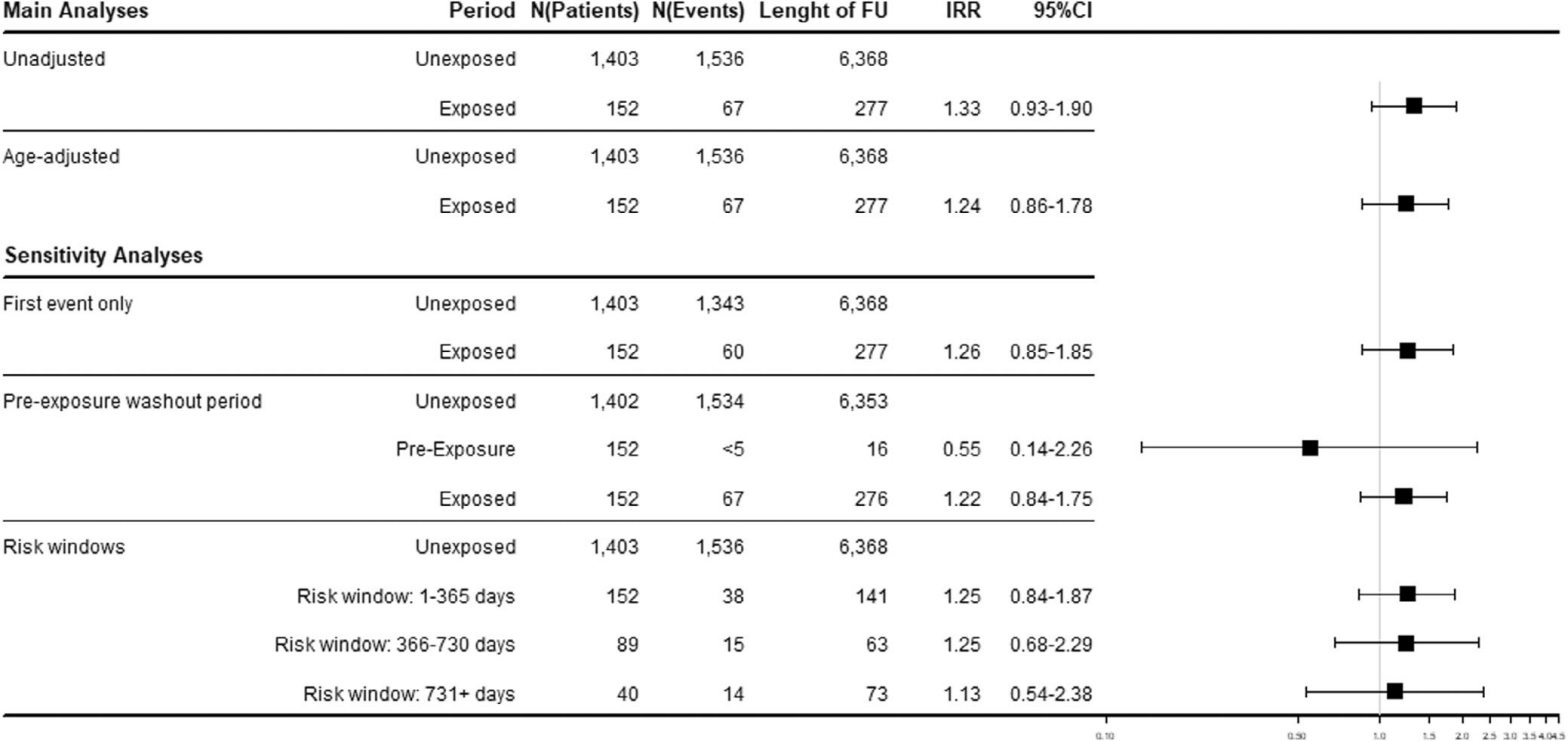
Results from SCCS analyses for GI ulcer (frailty cohort). BP = oral bisphosphonates; GI ulcer = gastrointestinal ulcer; HES = hospital episode statistics; IRR = incidence rate ratio.

Our SCCS models found no evidence of an association between BP exposure and incidence of GI ulcers, with IRR 1.24 (95% CI, 0.86–1.78) estimated from the age‐adjusted SCCS model (Fig. 3). Sensitivity analyses were consistent with that finding. Likewise, no significant associations were found for the hospitalization and polypharmacy cohorts (IRR 1.27 [95% CI, 0.85–1.90] and IRR 1.07 [95% CI, 0.76–1.49], respectively) (Table S4a/b/c).

## Discussion

### Principal findings

Our study found a 50% to 65% increased risk of severe AKI associated with BP use in elderly patients with complex health needs, as defined by polypharmacy, measurable frailty, or healthcare resource use. No evidence of an increased risk was found for upper GI ulcer in our patient cohorts.

### Severe AKI

Although AKI has been described in patients receiving high‐dose intravenous BPs, few studies have assessed nephrotoxicity associated with oral BP. Shih and colleagues^(^ ^21^ ^)^ assessed the risk of hospitalization for AKI in a population‐based cohort study among Canadian elderly patients, who started on oral BP after being discharged from hospital following a fragility fracture (mean age 81 years). Comparing risk for AKI within 90 days from treatment initiation, no increased risk was found compared to BP nonusers in multivariate logistic regression analysis (adjusted odds ratio [OR] 1.03; 95% CI, 0.84–1.26). In contrast, our study assessed AKI events during the whole course of patient follow‐up without restrictions to predefined time frames. Comparing patient baseline characteristics of both studies, more patients included in our study were diagnosed with chronic kidney disease compared to the Canadian study (60% versus ~30%). With underlying renal impairment, AKI risk may be increased, and accumulation of BP can occur, leading carryover effects when BP treatment is stopped. Oral BP are currently contraindicated in patients with severe renal impairment, as data on the safety of its use in patients with severe renal impairment was lacking. A recent CPRD‐based study among patients aged >40 years (mean age 80.3 years) with chronic kidney disease (CKD stage 3B+), found increased risk of CKD progression associated with BP use. However, no difference in risk for AKI was found comparing unexposed and BP‐exposed patients (hazard ratio [HR] 0.86; 95% CI, 0.67–1.09).^(^ ^22^ ^)^

This is the first study using the SCCS method to assess BP safety. Although the design was first applied in pharmacoepidemiological studies studying vaccine safety, it is increasingly being used in safety studies where confounding due to between‐person differences is of particular concern.^(^ ^15^, ^23^, ^24^, ^25^ ^)^ The SCCS approach relies on strong assumptions, and we assessed the robustness of our findings using sensitivity analyses. Multiple dependent events violate the assumption that outcomes studied in SCCS should be nonrecurrent or rare. However, with only 8% of patients having multiple AKI during follow‐up, recurrence of potentially dependent events was uncommon in our study. Our sensitivity analysis restricting to first AKI events only avoided that violation and showed similar results as the main analysis. In line with the literature reporting increased mortality risk associated with AKI,^(^ ^26^, ^27^, ^28^ ^)^ we found substantially higher mortality rates in patients with AKI. When restricting our data to patients who survived during follow‐up, the estimated IRR for AKI remained largely comparable to the main results, suggesting that although truncation of the observation period likely occurred in our study, its impact on the results remained small. Oral BP are contraindicated in patients with severe renal impairment; thus, initiation of BP might be postponed shortly after a patient experienced AKI. We introduced a 6‐month pre‐exposure washout period prior to the start of each exposed period to examine whether subsequent exposure depends on event occurrence or not. The duration of the pre‐exposed period was derived graphically. IRR for the exposed period was slightly increased when compared to our main model, which resulted from a reduced IR in the denominator when the pre‐exposure period was excluded from overall unexposed patient time. The increased risk for AKI (IRR 1.72; 95% CI, 1.22–2.44; Fig. 2) we found in the pre‐exposure period seems counterintuitive at the first glace, because it would be interpreted that AKI increases the probability of BP exposure. Although speculative, we interpret this finding to be due to confounding by indication, because severe fractures such as hip fractures were found to be associated with AKI: postoperative AKI was reported in a quarter of elderly patients hospitalized for hip fracture surgery.^(^ ^29^, ^30^ ^)^ Our sensitivity analysis excluding patients with any fracture within 1 year prior to first BP initiation showed consistent results with the main analysis. Although our study compared event rates during exposed and unexposed patient time, the actual timing of the events within the exposed periods was not assessed. However, as suggested by sensitivity analyses, patients might have stopped treatment when experiencing signs of AKI (Table S3c). As suggested by a reviewer comment, we conducted a post hoc analysis to stratify for gender (Table S3c, age adjustment using 5‐year age bands due to small sample size), which showed that increased risk might be more pronounced for men compared to women. This should be investigated further in future studies.

### GI ulcer

GI intolerance is among the most common extraskeletal BP side effects. With a very poor GI absorption of <1%, prolonged exposure of the GI mucosa to high BP concentrations could occur,^(^ ^31^ ^)^ carrying the risk for mucosa irritations. Because upper GI complications are frequently linked to improper administration of BPs, patients are advised to thoroughly follow the instructions for intake, namely to take BP on an empty stomach, with sufficient water, and to remain in an upright position for at least 30 minutes after intake,^(^ ^31^ ^)^ to minimize drug exposure to the esophagus.

The evidence of the association of GI complications and BP is inconclusive in the literature. Increased risk of upper GI bleeding (HR 1.32; 95% CI, 1.02–1.71) was associated with alendronate after adjusting for risk factors in a study based on Taiwan's National Health Insurance Research Database.^(^ ^32^ ^)^ Likewise, a population‐based cohort study in Canada found increased risk for upper GI bleeding in patients aged >80 years and individuals with a history of GI ulcer disease compared to younger patients in the first 4 months after BP treatment initiation.^(^ ^33^ ^)^ Although randomized clinical trials among postmenopausal osteoporotic women found high rates of upper GI tract events, incidences were similar for placebo and BP groups (alendronate^(^ ^34^ ^)^ or risedronate^(^ ^35^ ^)^). Similar findings were reported in a cohort study based on US claims data,^(^ ^36^ ^)^ which compared rates of GI events within 12 months before and after oral BP treatment initiation for osteoporotic women aged ≥55 (26.6% versus 28% for GI events; 3.6% versus 3.9% for severe GI events), highlighting the high background rates of GI events. Results from our study are in line with these findings, showing no increased ulcer risk during BP exposure compared to unexposed patient time. Although a case–control study among the elderly found no increased risk for upper GI bleeding associated with oral BP use alone, bleeding risk was increased in those using BP in combination with nonsteroidal anti‐inflammatory drugs (NSAIDs) (OR 2.0; 95% CI, 1.12–3.57)^(^ ^37^ ^)^ and in those using NSAIDs alone. Our study did not take co‐medication that may alter the risk for GI ulcer (eg, proton pump inhibitor [PPI] or NSAIDs) into account, because several substances from both drug classes can be purchased over the counter in the UK and may be missed in the data.

### Skeletal side effects

ONJ was very rare in the frailty cohort, with only 1.6 cases/100,000 person years. Incidence rates between 1 and 69/100,000 person years were reported in patients prescribed oral BPs.^(^ ^38^ ^)^

Fractures of the subtrochanteric/distal femur or femur shaft were assessed as proxies for “atypical” femur fracture (AFF) in line with a previous observational study, because no specific codes to identify radiographically confirmed AFF were available in CPRD. Previous studies highlighted that only a small proportion (between 3% and 13%) of overall subtrochanteric/distal/shaft femur fractures are classified as “atypical” after radiographic assessment.^(^ ^39^ ^)^ Therefore, incidence rates retrieved from this study are markedly higher compared to previous reports on AFF in BP users.^(^ ^40^ ^)^ However, rates of 3.4/1000 person‐years for subtrochanteric/ femur shaft fractures were described in alendronate users,^(^ ^39^ ^)^ which is about 2.6‐fold higher compared to our incidence rate in the frailty cohort.

### Limitations

Our study comes with strengths and limitations. We consider the use of the SCCS method a particular strength. SCCS accounts for patient‐level confounders such as frailty, which are difficult to assess from routinely collected data. Although we adjusted our analyses for age bands, other potential time‐varying confounders, such as development of new diseases or changes in exposure to co‐medication, were not captured. Therefore, future studies should further investigate the effect of time‐varying risk factors for BP‐associated adverse events in older people with complex health needs.

We did not adjust for changes in kidney function (eg, estimated glomerular filtration rate [eGFR]) during the study period. Measurements of eGFR may not be regularly collected for all patients but may be measured more regularly in patient at risk for kidney failure. eGFR may serve as a proxy for the outcome in addition.

CPRD data has been used extensively for pharmacoepidemiological studies. As for all prescription data, no information on actual drug dispensation and intake is available. Therefore, exposure misclassification cannot be ruled out. Persistence to BP was found to be relatively poor, with only ~30% of postmenopausal osteoporotic women continuing therapy for 2 years.^(^ ^41^ ^)^ However, among those who were persistent, compliance was high.

Outcomes were identified based on code lists used in previous studies, with the N17 code for AKI being previously validated.^(^ ^14^ ^)^ For GI ulcer, we identified events from both CPRD and HES to ensure events recorded in primary and secondary care settings were captured. Events recorded within 30 days from one another were removed to reduce re‐recordings of the same event. However, we only included main diagnosis for hospital admission—thus, AKI or GI ulcer recorded as secondary diagnoses were not included.

Our study focused specifically on BP safely in older patients with complex health needs, representing a particularly vulnerable and high‐risk population. Therefore, results cannot be generalized to the wider population of elderly BP users.

## Conclusions

BPs are commonly used in the elderly, but conflicting renal safety might trump their use in frail patients. Our study found a 50% to 65% increased risk for AKI associated with BP use in elderly patients with complex health needs. Although future studies should further investigate the risk–benefit of BP in susceptible patients, general practitioners (GPs) should consider to closely monitor kidney function in these frail patients when starting BP treatment.

## Author contributions

**Tetsuro Oda:** Conceptualization; formal analysis; methodology; writing – original draft; writing – review and editing. **Annika M. Jödicke:** Conceptualization; formal analysis; methodology; project administration; writing – original draft; writing – review and editing. **Danielle E. Robinson:** Conceptualization; methodology; supervision; writing – review and editing. **Antonella Delmestri:** Data curation; software; writing – review and editing. **Ruth H. Keogh:** Conceptualization; methodology; supervision; writing – review and editing. **Daniel Prieto‐Alhambra:** Conceptualization; funding acquisition; methodology; project administration; resources; supervision; writing – review and editing.

## Conflicts of Interest

DPA reports receiving grant support from Les Laboratoires Servier; that his research group has received grants and advisory or speaker fees from Amgen, Astellas, AstraZeneca, Chesi‐Taylor, Johnson and Johnson and UCD; and that Janssen, on behalf of Innovative Medicines Initiative‐funded European Health Data Evidence Network and European Medical Information Framework consortium and Synapse Management Partners, have supported training programs, open to external participants, organized by his department. TO reports employment with Chugai Pharmaceutical Co., Ltd. All other authors declare no conflict.

### Peer review

The peer review history for this article is available at https://publons.com/publon/10.1002/jbmr.4573.

## Ethical Approval

The study protocol was approved by the Independent Scientific Advisory Committee for MHRA Database Research (ISAC), including amendments (Protocol No 19_132A2) and will be made available to the journal reviewers upon request.

## Supporting information


**Table S1.** ICD‐10 and READ‐code lists for outcomes.
**Table S2.** Product code lists for oral bisphosphonates.

**Fig. S1.** Study design schema.
**Table S1a.** Patient demographics: Patients in hospitalization cohort with AKI or GI ulcer, respectively.
**Table S1b.** Patient demographics: Patients in polypharmacy cohort with AKI or GI ulcer, respectively.
**Table S2.** Incidence rates.
**Table S3a.** Results from SCCS analyses for severe AKI (hospitalization cohort).
**Table S3b.** Results from SCCS analyses for severe AKI (polypharmacy cohort).
**Table S3c.** Additional sensitivity analyses for severe AKI (all cohorts).
**Table S4a.** Results from SCCS analyses for GI ulcer (hospitalization cohort).
**Table S4b.** Results from SCCS analyses for GI ulcer (polypharmacy cohort).
**Table S4c.** Additional sensitivity analyses for GI ulcer (all cohorts).

## Data Availability

Data were obtained from CPRD under the Oxford University CPRD license. Direct data sharing is not allowed. Data access can be obtained from CPRD, conditional on ISAC approval.
