# Supplementary material for: Oral Bisphosphonates Are Associated With Increased Risk of Severe Acute Kidney Injury in Elderly Patients With Complex Health Needs: A Self‐Controlled Case Series in the United Kingdom
Source: J Bone Miner Res. 2022 Jun 8;37(7):1270–8. doi: 10.1002/jbmr.4573 (PMC9543096; doi:10.1002/jbmr.4573)
Supplement: Supplementary file 1 — Table S1. ICD‐10 and READ‐code lists for outcomes. Table S2. Product code lists for oral bisphosphonates. [file JBMR-37-1270-s002.docx]

**Supplement 1: Code lists**

# **Oral bisphosphonates are associated with increased risk of severe acute kidney injury in elderly patients with complex health needs: a self-controlled case series in the UK**

Table S1: ICD-10 and READ-code lists for outcomes

| ACUTE KIDNEY INJURY | |
| --- | --- |
| ICD-10 Code | **Description** |
| N17.0 | Acute renal failure with tubular necrosis |
| N17.1 | Acute renal failure with acute cortical necrosis |
| N17.2 | Acute renal failure with medullary necrosis |
| N17.8 | Other acute renal failure |
| N17.9 | Acute renal failure, unspecified |
| N19 | Unspecified kidney failure |
| UPPER GASTROINTESTINAL ULCER | |
| ICD-10 Code | **Description** |
| K25.0 | Gastric ulcer : acute with haemorrhage |
| K25.1 | Gastric ulcer : acute with perforation |
| K25.2 | Gastric ulcer : acute with both haemorrhage and perforation |
| K25.3 | Gastric ulcer : acute without haemorrhage or perforation |
| K25.4 | Gastric ulcer : chronic or unspecified with haemorrhage |
| K25.5 | Gastric ulcer : chronic or unspecified with perforation |
| K25.6 | Gastric ulcer : chronic or unspecified with both haemorrhage and perforation |
| K25.7 | Gastric ulcer : chronic without haemorrhage or perforation |
| K25.9 | Gastric ulcer : unspecified as acute or chronic, without haemorrhage or perforation |
| K26.0 | Duodenal ulcer : acute with haemorrhage |
| K26.1 | Duodenal ulcer : acute with perforation |
| K26.2 | Duodenal ulcer : acute with both haemorrhage and perforation |
| K26.3 | Duodenal ulcer : acute without haemorrhage or perforation |
| K26.4 | Duodenal ulcer : chronic or unspecified with haemorrhage |
| K26.5 | Duodenal ulcer : chronic or unspecified with perforation |
| K26.6 | Duodenal ulcer : chronic or unspecified with both haemorrhage and perforation |
| K26.7 | Duodenal ulcer : chronic without haemorrhage or perforation |
| K26.9 | Duodenal ulcer : unspecified as acute or chronic, without haemorrhage or perforation |
| K27.0 | Peptic ulcer, site unspecified : acute with haemorrhage |
| K27.1 | Peptic ulcer, site unspecified : acute with perforation |
| K27.2 | Peptic ulcer, site unspecified : acute with both haemorrhage and perforation |
| K27.3 | Peptic ulcer, site unspecified : acute without haemorrhage or perforation |
| K27.4 | Peptic ulcer, site unspecified : chronic or unspecified with haemorrhage |
| K27.5 | Peptic ulcer, site unspecified : chronic or unspecified with perforation |
| K27.6 | Peptic ulcer, site unspecified : chronic or unspecified with both haemorrhage and perforation |
| K27.7 | Peptic ulcer, site unspecified : chronic without haemorrhage or perforation |
| K27.9 | Peptic ulcer, site unspecified : unspecified as acute or chronic, without haemorrhage or perforation |
| K28.0 | Gastrojejunal ulcer : acute with haemorrhage |
| K28.1 | Gastrojejunal ulcer : acute with perforation |
| K28.2 | Gastrojejunal ulcer : acute with both haemorrhage and perforation |
| K28.3 | Gastrojejunal ulcer : acute without haemorrhage or perforation |
| K28.4 | Gastrojejunal ulcer : chronic or unspecified with haemorrhage |
| K28.5 | Gastrojejunal ulcer : chronic or unspecified with perforation |
| K28.6 | Gastrojejunal ulcer : chronic or unspecified with both haemorrhage and perforation |
| K28.7 | Gastrojejunal ulcer : chronic without haemorrhage or perforation |
| K28.9 | Gastrojejunal ulcer : unspecified as acute or chronic, without haemorrhage or perforation |
| READ-code | **READ-term** |
| J130.00 | Acute peptic ulcer |
| J130z00 | Acute peptic ulcer NOS |
| J130y00 | Acute peptic ulcer unspecified |
| J130100 | Acute peptic ulcer with haemorrhage |
| J130300 | Acute peptic ulcer with haemorrhage and perforation |
| J130200 | Acute peptic ulcer with perforation |
| J130000 | Acute peptic ulcer without mention of complication |
| J131.00 | Chronic peptic ulcer |
| J131z00 | Chronic peptic ulcer NOS |
| J131y00 | Chronic peptic ulcer unspecified |
| J131100 | Chronic peptic ulcer with haemorrhage |
| J131400 | Chronic peptic ulcer with obstruction |
| J131200 | Chronic peptic ulcer with perforation |
| J131000 | Chronic peptic ulcer without mention of complication |
| J13..00 | Peptic ulcer - (PU) site unspecified |
| J13z.00 | Peptic ulcer NOS |
| J102000 | Peptic ulcer of oesophagus |
| J13y.00 | Unspecified peptic ulcer |
| J13yz00 | Unspecified peptic ulcer NOS |
| J13y100 | Unspecified peptic ulcer with haemorrhage |
| J13y300 | Unspecified peptic ulcer with haemorrhage and perforation |
| J13y400 | Unspecified peptic ulcer with obstruction |
| J13y200 | Unspecified peptic ulcer with perforation |
| J13y000 | Unspecified peptic ulcer without mention of complication |
| J120.00 | Acute duodenal ulcer |
| J120z00 | Acute duodenal ulcer NOS |
| J120y00 | Acute duodenal ulcer unspecified |
| J120100 | Acute duodenal ulcer with haemorrhage |
| J120300 | Acute duodenal ulcer with haemorrhage and perforation |
| J120400 | Acute duodenal ulcer with obstruction |
| J120200 | Acute duodenal ulcer with perforation |
| J120000 | Acute duodenal ulcer without mention of complication |
| J110.00 | Acute gastric ulcer |
| J110z00 | Acute gastric ulcer NOS |
| J110y00 | Acute gastric ulcer unspecified |
| J110100 | Acute gastric ulcer with haemorrhage |
| J110300 | Acute gastric ulcer with haemorrhage and perforation |
| J110400 | Acute gastric ulcer with obstruction |
| J110200 | Acute gastric ulcer with perforation |
| J110000 | Acute gastric ulcer without mention of complication |
| J125.00 | Anti-platelet induced duodenal ulcer |
| J112.00 | Anti-platelet induced gastric ulcer |
| J112z00 | Anti-platelet induced gastric ulcer NOS |
| J110111 | Bleeding acute gastric ulcer |
| J121111 | Bleeding chronic duodenal ulcer |
| J111111 | Bleeding chronic gastric ulcer |
| J121.00 | Chronic duodenal ulcer |
| J121z00 | Chronic duodenal ulcer NOS |
| J121y00 | Chronic duodenal ulcer unspecified |
| J121100 | Chronic duodenal ulcer with haemorrhage |
| J121300 | Chronic duodenal ulcer with haemorrhage and perforation |
| J121400 | Chronic duodenal ulcer with obstruction |
| J121200 | Chronic duodenal ulcer with perforation |
| J121000 | Chronic duodenal ulcer without mention of complication |
| J111.00 | Chronic gastric ulcer |
| J111z00 | Chronic gastric ulcer NOS |
| J111y00 | Chronic gastric ulcer unspecified |
| J111100 | Chronic gastric ulcer with haemorrhage |
| J111300 | Chronic gastric ulcer with haemorrhage and perforation |
| J111400 | Chronic gastric ulcer with obstruction |
| J111200 | Chronic gastric ulcer with perforation |
| J111000 | Chronic gastric ulcer without mention of complication |
| J12..00 | Duodenal ulcer - (DU) |
| J122.00 | Duodenal ulcer disease |
| J12z.00 | Duodenal ulcer NOS |
| J11..00 | Gastric ulcer - (GU) |
| J11z.00 | Gastric ulcer NOS |
| J11z.12 | Multiple gastric ulcers |
| J126.00 | Non steroidal anti inflammatory drug induced duodenal ulcer |
| J113.00 | Non steroidal anti inflammatory drug induced gastric ulcer |
| J121211 | Perforated chronic duodenal ulcer |
| J111211 | Perforated chronic gastric ulcer |
| J124.00 | Recurrent duodenal ulcer |
| J12yy00 | Unspec duodenal ulcer; unspec haemorrhage and/or perforation |
| J11yy00 | Unspec gastric ulcer; unspec haemorrhage and/or perforation |
| J12y.00 | Unspecified duodenal ulcer |
| J12yz00 | Unspecified duodenal ulcer NOS |
| J12y100 | Unspecified duodenal ulcer with haemorrhage |
| J12y300 | Unspecified duodenal ulcer with haemorrhage and perforation |
| J12y400 | Unspecified duodenal ulcer with obstruction |
| J12y200 | Unspecified duodenal ulcer with perforation |
| J12y000 | Unspecified duodenal ulcer without mention of complication |
| J11y.00 | Unspecified gastric ulcer |
| J11yz00 | Unspecified gastric ulcer NOS |
| J11y100 | Unspecified gastric ulcer with haemorrhage |
| J11y400 | Unspecified gastric ulcer with obstruction |
| J11y200 | Unspecified gastric ulcer with perforation |
| J11y000 | Unspecified gastric ulcer without mention of complication |
| OSTEONECROSIS OF THE JAW | |
| ICD-10 Code | **Description** |
| K10.2 | Inflammatory conditions of jaws |
| READ-code | **READ-term** |
| J064200 | Chronic osteitis of jaw |
| J064500 | Chronic osteomyelitis of jaw |
| J064.11 | Osteitis - jaw |
| J064.12 | Osteomyelitis - jaw |
| 7J16000 | Osteotomy of alveolar segment of jaw NEC |
| 7J16800 | Osteotomy of alveolar segment of mandible |
| 7J16.13 | Osteotomy of jaw |
| 7J16600 | Osteotomy of mandible and advancement of mandible |
| 7J16700 | Osteotomy of mandible and retrusion of mandible |
| 7J16100 | Osteotomy of mandible NEC |
| 7J16A00 | Sagittal split mandibular osteotomy |
| 7J16B00 | Vertical sub-sigmoid mandibular osteotomy |
| FRACTURES OF THE SUBTROCHANTERIC/DISTAL FEMUR OR FEMUR SHAFT | |
| ICD-10 Code | **Description** |
| S72.4 | Fracture of lower end of femur |
| S72.2 | Subtrochanteric fracture |
| S72.3 | Fracture of shaft of femur |
| READ-code | **READ-term** |
| S312300 | Closed fracture distal femur, supracondylar |
| S312200 | Closed fracture of femur, lower epiphysis |
| S312.11 | Closed fracture of femur, distal end |
| S312.00 | Closed fracture distal femur |
| S4F4.00 | Closed fracture-dislocation, patello-femoral joint |
| S312500 | Closed fracture distal femur, lateral condyle |
| S4F6.00 | Closed fracture-subluxation, patello-femoral joint |
| S312400 | Closed fracture distal femur, medial condyle |
| S312000 | Closed fracture of distal femur, unspecified |
| S312600 | Closed fracture distal femur, bicondylar (T-Y fracture) |
| S312x00 | Closed fracture distal femur, comminuted/intra-articular |
| S312z00 | Closed fracture of distal femur not otherwise specified |
| S302200 | Closed fracture proximal femur, subtrochanteric |
| S310100 | Closed fracture shaft of femur |
| S314.00 | Fracture of shaft of femur |
| S305.00 | Subtrochanteric fracture |

Table S2: Product code lists for oral bisphosphonates

| Product code | DMD code | Substance name | Product name |
| --- | --- | --- | --- |
| 544 | 417211000001103 | Alendronate sodium | Fosamax Once Weekly 70mg tablets (Merck Sharp & Dohme Ltd) |
| 663 | 726311000001107 | Alendronate sodium | Fosamax 10mg tablets (Merck Sharp & Dohme Ltd) |
| 688 | 134599008 | Alendronate sodium | Alendronic acid 70mg tablets |
| 782 | 3145911000001101 | Alendronate sodium | Fosamax 5mg tablets (Merck Sharp & Dohme Ltd) |
| 2298 | 325974004 | Alendronate sodium | Alendronic acid 10mg tablets |
| 7530 | 325977006 | Alendronate sodium | Alendronic acid 5mg tablets |
| 35937 | 9221911000001101 | Alendronate sodium | Alendronic acid 70mg tablets (A A H Pharmace-inhibitorsuticals Ltd) |
| 37217 | 9251711000001102 | Alendronate sodium | Alendronic acid 10mg tablets (Teva UK Ltd) |
| 37218 | 9188811000001108 | Alendronate sodium | Alendronic acid 70mg tablets (Teva UK Ltd) |
| 40449 | 9836811000001109 | Alendronate sodium | Alendronic acid 70mg tablets (PLIVA Pharma Ltd) |
| 43958 | 9830711000001104 | Alendronate sodium | Alendronic acid 70mg tablets (Actavis UK Ltd) |
| 46245 | 9554311000001109 | Alendronate sodium | Alendronic acid 70mg tablets (Mylan) |
| 47380 | 10447611000001104 | Alendronate sodium | Alendronic acid 70mg tablets (Arrow Generics Ltd) |
| 50278 | 13441411000001109 | Alendronate sodium | Alendronic acid 70mg tablets (Wockhardt UK Ltd) |
| 50880 | 18264111000001108 | Alendronate sodium | Fosamax 10mg tablets (Necessity Supplies Ltd) |
| 51877 | 9299611000001102 | Alendronate sodium | Alendronic acid 70mg tablets (Alliance Healthcare (Distribution) Ltd) |
| 52284 | 14240211000001101 | Alendronate sodium | Fosamax 10mg tablets (Sigma Pharmace-inhibitorsuticals Plc) |
| 52624 | 17756411000001101 | Alendronate sodium | Alendronic acid 70mg tablets (Phoenix Healthcare Distribution Ltd) |
| 52834 | 18455911000001100 | Alendronate sodium | Alendronic acid 70mg tablets (Accord Healthcare Ltd) |
| 54566 | 9252411000001103 | Alendronate sodium | Alendronic acid 10mg tablets (A A H Pharmace-inhibitorsuticals Ltd) |
| 55965 | 9990311000001102 | Alendronate sodium | Alendronic acid 70mg tablets (Zentiva) |
| 56061 | 10435111000001104 | Alendronate sodium | Alendronic acid 10mg tablets (Actavis UK Ltd) |
| 56260 | 9208911000001102 | Alendronate sodium | Alendronic acid 70mg tablets (Kent Pharmace-inhibitorsuticals Ltd) |
| 56730 | 17963711000001100 | Alendronate sodium | Alendronic acid 70mg tablets (Almus Pharmace-inhibitorsuticals Ltd) |
| 57875 | 16181811000001107 | Alendronate sodium | Fosamax Once Weekly 70mg tablets (Lexon (UK) Ltd) |
| 58744 | 13880811000001102 | Alendronate sodium | Fosamax Once Weekly 70mg tablets (DE Pharmace-inhibitorsuticals) |
| 59079 | 17961911000001104 | Alendronate sodium | Alendronic acid 10mg tablets (Almus Pharmace-inhibitorsuticals Ltd) |
| 59247 | 18264311000001105 | Alendronate sodium | Fosamax Once Weekly 70mg tablets (Necessity Supplies Ltd) |
| 59485 | 18455711000001102 | Alendronate sodium | Alendronic acid 10mg tablets (Accord Healthcare Ltd) |
| 59555 | 9452511000001100 | Alendronate sodium | Alendronic acid 10mg tablets (Alliance Healthcare (Distribution) Ltd) |
| 61686 | 19701111000001102 | Alendronate sodium | Alendronic acid 70mg tablets (DE Pharmace-inhibitorsuticals) |
| 63008 | 24111211000001100 | Alendronate sodium | Alendronic acid 70mg tablets (Somex Pharma) |
| 63175 | 17756211000001100 | Alendronate sodium | Alendronic acid 10mg tablets (Phoenix Healthcare Distribution Ltd) |
| 64331 | 19700911000001106 | Alendronate sodium | Alendronic acid 10mg tablets (DE Pharmace-inhibitorsuticals) |
| 65008 | 30317811000001101 | Alendronate sodium | Alendronic acid 70mg effervescent tablets sugar free |
| 65905 | 9554111000001107 | Alendronate sodium | Alendronic acid 10mg tablets (Mylan) |
| 66203 | 30316711000001106 | Alendronate sodium | Binosto 70mg effervescent tablets (Internis Pharmace-inhibitorsuticals Ltd) |
| 69995 | 15631411000001101 | Alendronate sodium | Alendronic acid 35mg/ 5ml oral solution |
| 71851 | 15060811000001107 | Alendronate sodium | Alendronic acid 70mg tablets (Sigma Pharmace-inhibitorsuticals Plc) |
| 71963 | 15060411000001105 | Alendronate sodium | Alendronic acid 10mg tablets (Sigma Pharmace-inhibitorsuticals Plc) |
| 72541 | 15166911000001107 | Alendronate sodium | Alendronic acid 70mg/ 5ml oral solution |
| 73560 | 10688611000001108 | Alendronate sodium | Alendronic acid 10mg tablets (PLIVA Pharma Ltd) |
| 74859 | 5366911000001104 | Alendronate sodium | Fosamax Once Weekly 70mg tablets (Waymade Healthcare Plc) |
| 75094 | 21756211000001101 | Alendronate sodium | Alendronic acid 10mg tablets (Waymade Healthcare Plc) |
| 77297 |  | Alendronate sodium | Alendronic acid 70mg tablets (Focus Pharmace-inhibitorsuticals Ltd) |
| 45787 | 18683211000001101 | Alendronic acid | Alendronic acid 70mg/ 100ml oral solution unit dose sugar free |
| 52564 | 18680211000001106 | Alendronic acid | Alendronic acid 70mg/ 100ml oral solution unit dose sugar free (Rosemont Pharmace-inhibitorsuticals Ltd) |
| 55295 | 19185711000001106 | Alendronic acid | Alendronic acid 70mg/ 100ml oral solution unit dose sugar free (Alliance Healthcare (Distribution) Ltd) |
| 55998 | 20920711000001107 | Alendronic acid | Alendronic acid 70mg/ 75ml oral solution unit dose |
| 60144 | 21755811000001108 | Alendronic acid | Alendronic acid 70mg/ 100ml oral solution unit dose sugar free (Waymade Healthcare Plc) |
| 72208 | 20005911000001103 | Alendronic acid | Alendronic acid 70mg/ 100ml oral solution unit dose sugar free (A A H Pharmace-inhibitorsuticals Ltd) |
| 7224 | 9526611000001107 | Colecalciferol/ Alendronate sodium | Alendronic acid 70mg / Colecalciferol 70microgram tablets |
| 10227 | 9523811000001102 | Colecalciferol/ Alendronate sodium | Fosavance tablets (Merck Sharp & Dohme Ltd) |
| 66485 | 16182011000001109 | Colecalciferol/ Alendronate sodium | Fosavance tablets (Lexon (UK) Ltd) |
| 70927 | 34741911000001106 | Colecalciferol/ Alendronate sodium | Alendronic acid 70mg / Colecalciferol 70microgram tablets (Creo Pharma Ltd) |
| 76864 |  | Colecalciferol/ Alendronate sodium | Alendronic acid 70mg / Colecalciferol 140microgram tablets |
| 37575 | 249915001000027103 | Colecalciferol/ Risedronate Sodium/ Calcium Carbonate | Risedronate sodium 35mg with calcium carbonate 2500mg & colecalciferol 22micrograms tablets and granules |
| 53169 | 224555001000027100 | Disodium Etidronate | Etidronate disodium 400mg Tablet |
| 54436 | 247235001000027103 | Disodium Etidronate | Etidronate disodium Oral solution |
| 766 | 3356811000001108 | Etidronate disodium | Didronel 200mg tablets (Warner Chilcott UK Ltd) |
| 4680 | 325951003 | Etidronate disodium | Etidronate disodium 200mg tablets |
| 63371 | 5196211000001102 | Etidronate disodium | Etidronate disodium 200mg tablets (Mylan) |
| 77070 |  | Etidronate disodium | Etidronate disodium 100mg/ 5ml oral suspension |
| 77996 |  | Etidronate disodium | Didronel 400mg tablets (Mawdsley-Brooks & Company Ltd) |
| 110 | 151675001000027109 |  | DIDRONEL 100 MG TAB |
| 468 | 3352411000001105 |  | Didronel PMO tablets (Warner Chilcott UK Ltd) |
| 3046 | 81085001000027103 |  | DISODIUM ETIDRONATE 200 MG TAB |
| 11368 | 36133111000001109 |  | Calcium carbonate 1.25g effervescent tablets and Disodium etidronate 400mg tablets |
| 7112 | 9544911000001107 | Ibandronic sodium monohydrate | Bonviva 150mg tablets (Roche Products Ltd) |
| 7146 | 9553111000001105 | Ibandronic sodium monohydrate | Ibandronic acid 150mg tablets |
| 10193 | 410948006 | Ibandronic sodium monohydrate | Ibandronic acid 50mg tablets |
| 26913 | 7540111000001106 | Ibandronic sodium monohydrate | Bondronat 50mg tablets (Roche Products Ltd) |
| 47911 | 19371411000001104 | Ibandronic sodium monohydrate | Iasibon 50mg tablets (Aspire Pharma Ltd) |
| 51342 | 13837111000001101 | Ibandronic sodium monohydrate | Bonviva 150mg tablets (DE Pharmace-inhibitorsuticals) |
| 54453 | 19866411000001102 | Ibandronic sodium monohydrate | Bonviva 150mg tablets (Lexon (UK) Ltd) |
| 56030 | 20641211000001107 | Ibandronic sodium monohydrate | Ibandronic acid 150mg tablets (A A H Pharmace-inhibitorsuticals Ltd) |
| 56369 | 22086511000001105 | Ibandronic sodium monohydrate | Ibandronic acid 150mg tablets (Zentiva) |
| 57980 | 19295711000001108 | Ibandronic sodium monohydrate | Ibandronic acid 50mg tablets (Actavis UK Ltd) |
| 59587 | 23166211000001109 | Ibandronic sodium monohydrate | Ibandronic acid 150mg tablets (Ranbaxy (UK) Ltd) |
| 67159 | 20596011000001106 | Ibandronic sodium monohydrate | Ibandronic acid 50mg tablets (Teva UK Ltd) |
| 71000 | 20971311000001104 | Ibandronic sodium monohydrate | Ibandronic acid 150mg tablets (Alliance Healthcare (Distribution) Ltd) |
| 75425 |  | Ibandronic sodium monohydrate | Ibandronic acid 50mg tablets (DE Pharmace-inhibitorsuticals) |
| 75644 |  | Ibandronic sodium monohydrate | Quodixor 150mg tablets (Aspire Pharma Ltd) |
| 76545 |  | Ibandronic sodium monohydrate | Bonviva 150mg tablets (Mawdsley-Brooks & Company Ltd) |
| 77225 |  | Ibandronic sodium monohydrate | Ibandronic acid 150mg tablets (Teva UK Ltd) |
| 78002 |  | Ibandronic sodium monohydrate | Ibandronic acid 150mg tablets (Mylan) |
| 6058 | 408027002 | Risedronate sodium | Risedronate sodium 35mg tablets |
| 6084 | 215955001000027104 | Risedronate sodium | Actonel once a week 35mg Tablet (Procter & Gamble (Health & Beauty Care) Ltd) |
| 6634 | 325983009 | Risedronate sodium | Risedronate sodium 5mg tablets |
| 7089 | 325984003 | Risedronate sodium | Risedronate sodium 30mg tablets |
| 7527 | 892511000001105 | Risedronate sodium | Actonel 5mg tablets (Warner Chilcott UK Ltd) |
| 7546 | 3778711000001100 | Risedronate sodium | Actonel 30mg tablets (Warner Chilcott UK Ltd) |
| 44511 | 4028511000001107 | Risedronate sodium | Actonel Once a Week 35mg tablets (Warner Chilcott UK Ltd) |
| 48013 | 18448311000001108 | Risedronate sodium | Risedronate sodium 35mg tablets (A A H Pharmace-inhibitorsuticals Ltd) |
| 52373 | 18626011000001106 | Risedronate sodium | Risedronate sodium 35mg tablets (Phoenix Healthcare Distribution Ltd) |
| 56431 | 18597911000001107 | Risedronate sodium | Risedronate sodium 35mg tablets (Actavis UK Ltd) |
| 56663 | 21887311000001101 | Risedronate sodium | Risedronate sodium 35mg tablets (Waymade Healthcare Plc) |
| 58618 | 16131211000001104 | Risedronate sodium | Risedronate sodium 35mg/ 5ml oral solution |
| 59449 | 19215711000001107 | Risedronate sodium | Risedronate sodium 35mg tablets (Bluefish Pharmace-inhibitorsuticals AB) |
| 59916 | 20889411000001109 | Risedronate sodium | Risedronate sodium 35mg tablets (Sandoz Ltd) |
| 60288 | 16255311000001105 | Risedronate sodium | Actonel Once a Week 35mg tablets (Mawdsley-Brooks & Company Ltd) |
| 61313 | 18448111000001106 | Risedronate sodium | Risedronate sodium 30mg tablets (A A H Pharmace-inhibitorsuticals Ltd) |
| 63802 | 19863711000001101 | Risedronate sodium | Actonel Once a Week 35mg tablets (Lexon (UK) Ltd) |
| 64431 | 18359911000001105 | Risedronate sodium | Risedronate sodium 30mg tablets (Aspire Pharma Ltd) |
| 65971 | 16131311000001107 | Risedronate sodium | Risedronate sodium 35mg/ 5ml oral suspension |
| 66028 | 13211011000001106 | Risedronate sodium | Actonel 35mg tablets (Teva UK Ltd) |
| 67078 | 18308311000001108 | Risedronate sodium | Risedronate sodium 35mg tablets (Teva UK Ltd) |
| 69630 | 20536311000001101 | Risedronate sodium | Risedronate sodium 35mg tablets (Almus Pharmace-inhibitorsuticals Ltd) |
| 69929 | 18344211000001102 | Risedronate sodium | Risedronate sodium 35mg tablets (Alliance Healthcare (Distribution) Ltd) |
| 69958 | 33614811000001104 | Risedronate sodium | Risedronate sodium 35mg tablets (Mylan) |
| 71209 | 30879111000001108 | Risedronate sodium | Risedronate sodium 35mg tablets (Mawdsley-Brooks & Company Ltd) |
| 73454 | 18359411000001102 | Risedronate sodium | Risedronate sodium 35mg tablets (Aspire Pharma Ltd) |
| 73989 | 13098211000001102 | Risedronate sodium | Actonel Once a Week 35mg tablets (Dowelhurst Ltd) |
| 74805 | 13823311000001109 | Risedronate sodium | Actonel Once a Week 35mg tablets (DE Pharmace-inhibitorsuticals) |
| 76178 |  | Risedronate sodium | Actonel 5mg tablets (Sigma Pharmace-inhibitorsuticals Plc) |
| 76190 |  | Risedronate sodium | Risedronate sodium 5mg tablets (A A H Pharmace-inhibitorsuticals Ltd) |
| 45280 | 247875001000027107 | Risedronate Sodium/ Calcium Carbonate | Risedronate sodium 35mg & calcium carbonate 1250mg tablet |
| 37833 | 13208811000001109 |  | Actonel Combi 35mg tablets and 1000mg/ 880unit effervescent granules sachets (Teva UK Ltd) |
| 3680 | 3848211000001108 | Sodium clodronate | Loron 400mg capsules (Roche Products Ltd) |
| 4868 | 325965001 | Sodium clodronate | Sodium clodronate 400mg capsules |
| 4927 | 3847911000001100 | Sodium clodronate | Bonefos 400mg capsules (Bayer Plc) |
| 5629 | 325972000 | Sodium clodronate | Sodium clodronate 800mg tablets |
| 6568 | 325970008 | Sodium clodronate | Sodium clodronate 520mg tablets |
| 9189 | 920611000001107 | Sodium clodronate | Bonefos 800mg tablets (Bayer Plc) |
| 11244 | 3813811000001107 | Sodium clodronate | Loron 520mg tablets (Intrapharm Laboratories Ltd) |
| 39043 | 11550411000001104 | Sodium clodronate | Clasteon 400mg capsules (Kent Pharmace-inhibitorsuticals Ltd) |
| 54989 | 20540511000001106 | Sodium clodronate | Clasteon 800mg tablets (Beacon Pharmace-inhibitorsuticals Ltd) |
| 9208 | 325979009 | Tiludronate disodium | Tiludronic acid 200mg tablets |
| 9525 | 4122511000001103 | Tiludronate disodium | Skelid 200mg tablets (Sanofi) |
