# Supplementary material for: Oral Bisphosphonates Are Associated With Increased Risk of Severe Acute Kidney Injury in Elderly Patients With Complex Health Needs: A Self‐Controlled Case Series in the United Kingdom
Source: J Bone Miner Res. 2022 Jun 8;37(7):1270–8. doi: 10.1002/jbmr.4573 (PMC9543096; doi:10.1002/jbmr.4573)
Supplement: Supplementary file 2 — Fig. S1. Study design schema. Table S1a. Patient demographics: Patients in hospitalization cohort with AKI or GI ulcer, respectively. Table S1b. Patient demographics: Patients in polypharmacy cohort with AKI or GI ulcer, respectively. Table S2. Incidence rates. Table S3a. Results from SCCS analyses for severe AKI (hospitalization cohort). Table S3b. Results from SCCS analyses for severe AKI (polypharmacy cohort). Table S3c. Additional sensitivity analyses for severe AKI (all cohorts). Table S4a. Results from SCCS analyses for GI ulcer (hospitalization cohort). Table S4b. Results from SCCS analyses for GI ulcer (polypharmacy cohort). Table S4c. Additional sensitivity analyses for GI ulcer (all cohorts). [file JBMR-37-1270-s001.docx]

**Supplement 2**

# **Oral bisphosphonates are associated with increased risk of severe acute kidney injury in elderly patients with complex health needs: a self-controlled case series in the UK**

Figure S1: Study design schema

Baseline characteristics assessment

Study start (01/01/2010)

End of follow-up

Unexposed time

BP exposed time

Unexposed time

time

Table S1a: Patient demographics: Patients in hospitalisation cohort with AKI or GI ulcer, respectively

| **Characteristic** | **AKI** | | **GI ulcer** | |
| --- | --- | --- | --- | --- |
|  | Exposed | Unexposed | Exposed | Unexposed |
| **N** | 197 | 1,753 | 128 | 891 |
| **Age**, years | 80 [74, 85)] | 80 [74, 86] | 78 [74, 83] | 77 [71, 83] |
| **Gender** [Female] | 116 (59%) | 786 (45%) | 83 (65%) | 394 (44%) |
| **Follow-up,** years | 4.92 [3.52, 6.55] | 3.82 [1.96, 5.63] | 5.4 [3.91, 6.93] | 4.47 [2.64, 6.31] |
| **Total treatment duration,** days | 483 [194, 1,006] | NA | 470 [235, 892] | NA |
| **Number of treatment episodes** |  |  |  |  |
| 0 | 0 (0%) | 1,753 (100%) | 0 (0%) | 891 (100%) |
| 1 | 158 (80%) | 0 (0%) | 97 (76%) | 0 (0%) |
| 2 | 29 (15%) | 0 (0%) | 23 (18%) | 0 (0%) |
| ≥3 | 10 (5.1%) | 0 (0%) | 8 (6.3%) | 0 (0%) |
| **Number of respective outcome events*** |  |  |  |  |
| 1 | 182 (92%) | 1,611 (92%) | 114 (89%) | 803 (90%) |
| 2 | 14 (7.1%) | 122 (7.0%) | 11 (8.6%) | 74 (8.3%) |
| 3 | 1 (0.5%) | 17 (1.0%) | <5 | 12 (1.3%) |
| ≥4 | 0 (0%) | <5 | <5 | <5 |
| **Index of Multiple Deprivation** |  |  |  |  |
| 1 (most deprived) | 51 (26%) | 361 (21%) | 45 (35%) | 186 (21%) |
| 2 | 32 (16%) | 400 (23%) | 23 (18%) | 206 (23%) |
| 3 | 42 (21%) | 377 (22%) | 19 (15%) | 189 (21%) |
| 4 | 41 (21%) | 346 (20%) | 21 (16%) | 177 (20%) |
| 5 (least deprived) | 31 (16%) | 267 (15%) | 20 (16%) | 132 (15%) |
| missing | 0 (0%) | <5 | 0 (0%) | <5 |
| **Body Mass Index** |  |  |  |  |
| Underweight | 6 (3.0%) | 21 (1.2%) | <5 | 12 (1.3%) |
| Normal | 48 (24%) | 414 (24%) | 49 (38%) | 234 (26%) |
| Overweight | 52 (26%) | 510 (29%) | 37 (29%) | 293 (33%) |
| Obese | 63 (32%) | 511 (29%) | 30 (23%) | 215 (24%) |
| Missing | 28 (14%) | 297 (17%) | 11 (8.6%) | 137 (15%) |
| **Drinking status (5yr)** |  |  |  |  |
| Drinker | 16 (8.2%) | 114 (6.5%) | <5 | 83 (9.3%) |
| Ex-drinker | 63 (32%) | 553 (32%) | 42 (33%) | 271 (31%) |
| Non-drinker | 49 (25%) | 507 (29%) | 43 (34%) | 248 (28%) |
| Missing | 69 (35%) | 579 (33%) | 41 (32%) | 289 (32%) |
| **Smoking status (5yr)** |  |  |  |  |
| Smoker | 19 (9.6%) | 157 (9.0%) | <5 | 118 (13%) |
| Ex-smoker | 90 (46%) | 778 (44%) | 58 (45%) | 374 (42%) |
| Non-smoker | 79 (40%) | 736 (42%) | 60 (47%) | 349 (39%) |
| Missing | 9 (4.6%) | 82 (4.7%) | 6 (4.7%) | 50 (5.6%) |
| **Number of different drugs (1yr)** |  |  |  |  |
| <5 | 10 (5.1%) | 145 (8.3%) | 16 (12%) | 136 (15%) |
| 5-9 | 51 (26%) | 509 (29%) | 30 (23%) | 314 (35%) |
| 10-15 | 73 (37%) | 581 (33%) | 45 (35%) | 245 (27%) |
| >15 | 63 (32%) | 518 (30%) | 37 (29%) | 196 (22%) |
| **Charlson Comorbidity Index (1yr)** |  |  |  |  |
| 0 | 144 (73%) | 1,226 (70%) | 107 (84%) | 671 (75%) |
| 1 | 25 (13%) | 214 (12%) | 14 (11%) | 116 (13%) |
| 2 | 25 (13%) | 215 (12%) | 6 (4.7%) | 68 (7.6%) |
| ≥3 | <5 | 98 (5.6%) | <5 | 36 (4.0%) |
| **eFI (1yr)** | 3 [2, 4] | 3 [2, 4] | 2 [2, 4] | 2 [1, 4] |
| **GP visits** | 15 [8, 23] | 14 [9, 23] | 14 [8, 23] | 13 [8, 21] |
| **Fracture before BP initiation (1yr)** | 71 (36%) | NA | 51 (40%) | NA |
| **History of respective outcome*** | 17 (8.6%) | 147 (8.4%) | 10 (7.8%) | 96 (11%) |
| **History of osteoporosis**** | 10 (5.1%) | 60 (3.4%) | 13 (10%) | 23 (2.6%) |
| **History of chronic renal impairment**** | 88 (45%) | 883 (50%) | 95 (74%) | 621 (70%) |
| **Death** [Died] | 105 (53%) | 1,066 (61%) | 49 (38%) | 364 (41%) |

Statistics presented: Median [IQR]; n (%),

eFI = electronic frailty index, GP = general practitioner, BP = oral bisphosphonates, NA = treatment duration and date of BP initiation not available for BP unexposed people

*AKI events for AKI cohort, GI ulcer event for GI ulcer cohort, **anytime in patient history

Note: For each person, the most recent recording within 5 years prior to study start was considered for BMI, drinking status and smoking status, or labelled “missing” of no recording was available in that timeframe. Number of different drugs, number of GP visits Charlson Comorbidity index, eFI were calculated based on the year before study start. Fracture before BP initiation (yes/no) was calculated based on the year before the first BP prescription for people with BP prescriptions.

Table S1b: Patient demographics: Patients in polypharmacy cohort with AKI or GI ulcer, respectively

| **Characteristic** | **AKI** | | **GI ulcer** | |
| --- | --- | --- | --- | --- |
|  | Exposed | Unexposed | Exposed | Unexposed |
| **N** | 336 | 2,656 | 194 | 1,259 |
| **Age**, years | 79 [74, 84] | 79 [74, 85] | 77 [72, 81] | 77 [72, 82] |
| **Gender** [Female] | 241 (72%) | 1,238 (47%) | 121 (62%) | 616 (49%) |
| **Follow-up,** years | 5.62 [3.79, 7.13] | 4.18 [2.38, 6.03] | 5.85 [4.23, 7.65] | 4.76 [3.04, 6.55] |
| **Total treatment duration,** days | 528 [198, 1,070] | NA | 530 [264, 1,038] | NA |
| **Number of treatment episodes** |  |  |  |  |
| 0 | 0 (0%) | 2,656 (100%) | 0 (0%) | 1,259 (100%) |
| 1 | 265 (79%) | 0 (0%) | 145 (75%) | 0 (0%) |
| 2 | 51 (15%) | 0 (0%) | 34 (18%) | 0 (0%) |
| ≥3 | 20 (6.0%) | 0 (0%) | 15 (7.7%) | 0 (0%) |
| **Number of respective outcome events*** |  |  |  |  |
| 1 | 309 (92%) | 2,433 (92%) | 179 (92%) | 1,112 (88%) |
| 2 | 21 (6.2%) | 194 (7.3%) | 12 (6.2%) | 120 (9.5%) |
| 3 | 6 (1.8%) | 26 (1.0%) | <5 | 20 (1.6%) |
| ≥4 | 0 (0%) | <5 | <5 | 7 (0.4%) |
| **Index of Multiple Deprivation** |  |  |  |  |
| 1 (most deprived) | 78 (23%) | 532 (20%) | 49 (25%) | 259 (21%) |
| 2 | 58 (17%) | 592 (22%) | 44 (23%) | 299 (24%) |
| 3 | 78 (23%) | 554 (21%) | 31 (16%) | 246 (20%) |
| 4 | 75 (22%) | 569 (21%) | 37 (19%) | 251 (20%) |
| 5 (least deprived) | 47 (14%) | 407 (15%) | 33 (17%) | 203 (16%) |
| missing | 0 (0%) | <5 | 0 (0%) | <5 |
| **Body Mass Index** |  |  |  |  |
| Underweight | 8 (2.4%) | 29 (1.1%) | <5 | 13 (1.0%) |
| Normal | 81 (24%) | 514 (19%) | 62 (32%) | 254 (20%) |
| Overweight | 95 (28%) | 819 (31%) | 56 (29%) | 454 (36%) |
| Obese | 127 (38%) | 1,012 (38%) | 48 (25%) | 409 (32%) |
| Missing | 25 (7.4%) | 282 (11%) | 25 (13%) | 129 (10%) |
| **Drinking status (5yr)** |  |  |  |  |
| Drinker | 24 (7.2%) | 148 (5.6%) | 13 (6.7%) | 106 (8.5%) |
| Ex-drinker | 98 (29%) | 936 (35%) | 62 (32%) | 440 (35%) |
| Non-drinker | 104 (31%) | 775 (29%) | 59 (31%) | 357 (29%) |
| Missing | 110 (33%) | 797 (30%) | 60 (31%) | 356 (28%) |
| **Smoking status (5yr)** |  |  |  |  |
| Smoker | 31 (9.2%) | 204 (7.7%) | 19 (9.8%) | 143 (11%) |
| Ex-smoker | 145 (43%) | 1,287 (48%) | 82 (42%) | 585 (46%) |
| Non-smoker | 151 (45%) | 1,088 (41%) | 90 (46%) | 490 (39%) |
| Missing | 9 (2.7%) | 77 (2.9%) | <5 | 41 (3.3%) |
| **Number of different drugs (1yr)** |  |  |  |  |
| 10-15 | 210 (62%) | 1,669 (63%) | 69 (36%) | 399 (32%) |
| >15 | 126 (38%) | 987 (37%) | 125 (64%) | 860 (68%) |
| **Charlson Comorbidity Index (1yr)** |  |  |  |  |
| 0 | 254 (76%) | 1,903 (72%) | 160 (82%) | 969 (77%) |
| 1 | 37 (11%) | 271 (10%) | 18 (9.3%) | 126 (10%) |
| 2 | 39 (12%) | 343 (13%) | 12 (6.2%) | 119 (9.5%) |
| ≥3 | 6 (1.8%) | 139 (5.2%) | <5 | 45 (3.6%) |
| **eFI (1yr)** | 3 [2, 4] | 3 [2, 4] | 3 [2, 3] | 3 [2, 4] |
| **GP visits** | 16 [10, 23] | 15 [10, 23] | 16 [10, 23] | 15 [10, 22] |
| **Fracture before BP initiation (1yr)** | 107 (32%) | NA | 68 (35%) | NA |
| **History of respective outcome*** | 17 (5.1%) | 162 (6.1%) | 13 (6.7%) | 110 (8.7%) |
| **History of osteoporosis**** | 14 (4.2%) | 98 (3.7%) | 13 (6.7%) | 33 (2.6%) |
| **History of chronic renal impairment**** | 157 (47%) | 1,541 (58%) | 135 (70%) | 780 (62%) |
| **Death** [Died] | 175 (52%) | 1,622 (61%) | 69 (36%) | 477 (38%) |

Statistics presented: Median [IQR]; n (%),

eFI = electronic frailty index, GP = general practitioner, BP = oral bisphosphonates, NA = treatment duration and date of BP initiation not available for BP unexposed people

*AKI events for AKI cohort, GI ulcer event for GI ulcer cohort, **anytime in patient history

Note: For each person, the most recent recording within 5 years prior to study start was considered for BMI, drinking status and smoking status, or labelled “missing” of no recording was available in that timeframe. Number of different drugs, number of GP visits Charlson Comorbidity index, eFI were calculated based on the year before study start. Fracture before BP initiation (yes/no) was calculated based on the year before the first BP prescription for people with BP prescriptions

Table S2: Incidence rates

| **Analysis** | | **N(events)** | | **Person years** | | **IR/100,000 py** | | **95%CI** | |
| --- | --- | --- | --- | --- | --- | --- | --- | --- | --- |
| **Frailty cohort** |  | |  | |  | |  | |  |
| AKI | | 2835 | | 366,157.5 | | 774.3 | | 745.8, 802.8 | |
| GI ulcer | | 1271 | | 358,104.7 | | 354.9 | | 335.4, 374.4 | |
| Osteonecrosis of the jaw | | 6 | | 375,033.7 | | 1.6 | | 0.3, 4.9 | |
| Femur fractures | | 482 | | 373,565.8 | | 129.0 | | 117.5, 140.6 | |
| **Hospitalisation cohort** | | | | |  | |  | |  |
| AKI | | 1786 | | 298,650.1 | | 598.0 | | 570.3, 625.8 | |
| GI ulcer | | 913 | | 292,420.2 | | 312.2 | | 292.0, 332.5 | |
| Osteonecrosis of the jaw | | 9 | | 305,297.7 | | 3.0 | | 1.0, 4.9 | |
| Femur fractures | | 356 | | 303,960.0 | | 117.1 | | 105.0, 129.3 | |
| **Polypharmacy cohort** | | |  | |  | |  | |  |
| AKI | | 2813 | | 386,038.4 | | 728.7 | | 701.8, 755.6 | |
| GI ulcer | | 1330 | | 375,721.7 | | 354.0 | | 335.0, 373.0 | |
| Osteonecrosis of the jaw | | 8 | | 394,820.1 | | 2.0 | | 0.6, 3.4 | |
| Femur fractures | | 461 | | 393,270.6 | | 117.2 | | 106.5, 127.9 | |

N(events) = Number of events, py = person years, 95%CI = 95% Confidence Interval, AKI = severe acute kidney injury, GI ulcer = gastrointestinal ulcer

Table S3a: Results from SCCS analyses for severe AKI (hospitalisation cohort)

| **Analysis** | **Period** | **N(patients)** | **N(events)** | **Length of FU** | **IRR** | **95%CI** |
| --- | --- | --- | --- | --- | --- | --- |
| **Main analyses** |  |  |  |  |  |  |
| Unadjusted model | Unexposed | 1,950 | 2,022 | 7,592 |  |  |
|  | Exposed | 197 | 109 | 368 | 2.272 | 1.636, 3.155 |
| Age-adjusted model | Unexposed | 1,950 | 2,022 | 7,592 |  |  |
|  | Exposed | 197 | 109 | 368 | 1.495 | 1.052, 2.123 |
| **Sensitivity analyses** | | | | | | |
| First event only | Unexposed | 1,950 | 1,847 | 7,592 |  |  |
|  | Exposed | 197 | 103 | 368 | 1.479 | 1.027, 2.131 |
| Alive during study | Unexposed | 779 | 803 | 3,836 |  |  |
|  | Exposed | 92 | 55 | 214 | 1.861 | 1.135, 3.051 |
| Pre-exposure washout period | Unexposed | 1,936 | 1,990 | 7,487 |  |  |
|  | Pre-exposure | 197 | 37 | 115 | 1.712 | 1.119, 2.619 |
|  | Exposed | 197 | 109 | 368 | 1.765 | 1.21, 2.574 |
| Risk windows | Unexposed | 1,950 | 2,022 | 7,592 |  |  |
|  | Risk window:  1-365 days | 197 | 52 | 178 | 1.412 | 0.966, 2.064 |
|  | Risk window:  366-730 days | 106 | 24 | 86 | 1.653 | 0.936, 2.919 |
|  | Risk window:  731+ days | 63 | 33 | 104 | 1.929 | 0.987, 3.772 |

IRR = Incidence rate ratio, 95%CI = 95% Confidence Interval, Length of follow-up (FU) is provided in person-years

Table S3b: Results from SCCS analyses for severe AKI (polypharmacy cohort)

| **Analysis** | **Period** | **N(patients)** | **N(events)** | **Length of FU** | **IRR** | **95%CI** |
| --- | --- | --- | --- | --- | --- | --- |
| **Main analyses** |  |  |  |  |  |  |
| Unadjusted model | Unexposed | 2,992 | 3,095 | 12,653 |  |  |
|  | Exposed | 336 | 187 | 659 | 2.463 | 1.924, 3.155 |
| Age-adjusted model | Unexposed | 2,992 | 3,095 | 12,653 |  |  |
|  | Exposed | 336 | 187 | 659 | 1.592 | 1.219, 2.079 |
| **Sensitivity analyses** | | |  |  |  |  |
| First event only | Unexposed | 2,992 | 2,820 | 12,653 |  |  |
|  | Exposed | 336 | 172 | 659 | 1.582 | 1.199, 2.089 |
| Alive during study | Unexposed | 1,195 | 1,215 | 6,277 |  |  |
|  | Exposed | 161 | 90 | 364 | 1.642 | 1.146, 2.353 |
| Pre-exposure washout period | Unexposed | 2,975 | 3,043 | 12,468 |  |  |
|  | Pre-exposure | 336 | 61 | 205 | 1.837 | 1.326, 2.546 |
|  | Exposed | 336 | 187 | 659 | 1.887 | 1.42, 2.508 |
| Risk windows | Unexposed | 2,992 | 3,095 | 12,653 |  |  |
|  | Risk window:  1-365 days | 336 | 96 | 315 | 1.655 | 1.247, 2.197 |
|  | Risk window:  366-730 days | 190 | 35 | 151 | 1.34 | 0.862, 2.084 |
|  | Risk window:  731+ days | 108 | 56 | 193 | 1.555 | 0.948, 2.549 |

IRR = Incidence rate ratio, 95%CI = 95% Confidence Interval, Length of follow-up (FU) is provided in person-years

Table S3c: Additional sensitivity analyses for severe AKI (all cohorts)

| **Analysis** | | **Period** | | **N(patients)** | | **N(events)** | | **Length of FU** | | **IRR** | | **95%CI** | |
| --- | --- | --- | --- | --- | --- | --- | --- | --- | --- | --- | --- | --- | --- |
| **Frailty cohort** | |  | |  | |  | |  | |  | |  | |
| BP gap length 180days | | Unexposed | | 3,023 | | 3,132 | | 12,686 | |  | |  | |
|  |  | Exposed | | 307 | | 171 | | 547 | | 1.625 | | 1.229, 2.149 | |
| AKI: ICD-10 N17 | | Unexposed | | 2,962 | | 3,059 | | 12,504 | |  | |  | |
|  | | Exposed | | 299 | | 167 | | 530 | | 1.653 | | 1.245, 2.195 | |
| No fracture history | | Unexposed | | 2,922 | | 3,074 | | 12,338 | |  | |  | |
|  | | Exposed | | 206 | | 116 | | 373 | | 1.973 | | 1.406, 2.77 | |
| No 90days washout included for exposed period | | Unexposed | | 3,023 | | 3,169 | | 12,747 | |  | |  | |
|  |  | Exposed | | 307 | | 134 | | 486 | | 1.128 | | 0.842, 1.511 | |
| No 90days washout included for exposed period, but added separately after each exposed period* | | Unexposed | | 3,023 | | 3,132 | | 12,690 | |  | |  | |
|  |  | Exposed | | 307 | | 134 | | 486 | | 1.364 | | 1.005, 1.85 | |
|  |  | Washout | | 225 | | 37 | | 57 | | 2.723 | | 1.85, 4.008 | |
| Stratification for gender** | |  | |  | |  | |  | |  | |  | |
| Females | | Unexposed | | 1,430 | | 1,455 | | 5,998 | |  | |  | |
|  | | Exposed | | 213 | | 109 | | 408 | | 1.513* | | 1.093, 2.094* | |
| Males | | Unexposed | | 1,593 | | 1,677 | | 6,692 | |  | |  | |
|  | | Exposed | | 94 | | 62 | | 135 | | 4.049* | | 2.537, 6.463* | |
| **Hospitalisation cohort** | |  | |  | |  | |  | |  | |  | |
| BP gap length 180days | | Unexposed | | 1,950 | | 2,022 | | 7,589 | |  | |  | |
|  |  | Exposed | | 197 | | 109 | | 370 | | 1.469 | | 1.034, 2.088 | |
| AKI: ICD-10 N17 | | Unexposed | | 1,900 | | 1,963 | | 7,435 | |  | |  | |
|  | | Exposed | | 192 | | 106 | | 361 | | 1.48 | | 1.035, 2.116 | |
| No fracture history | | Unexposed | | 1,879 | | 1,983 | | 7,357 | |  | |  | |
|  | | Exposed | | 126 | | 70 | | 232 | | 1.637 | | 1.069, 2.508 | |
| No 90days washout included for exposed period | | Unexposed | | 1,950 | | 2,039 | | 7,629 | |  | |  | |
|  |  | Exposed | | 197 | | 92 | | 330 | | 1.288 | | 0.892, 1.858 | |
| No 90days washout included for exposed period, but added separately after each exposed period* | | Unexposed | | 1,950 | | 2,022 | | 7,592 | |  | |  | |
|  |  | Exposed | | 197 | | 92 | | 330 | | 1.415 | | 0.968, 2.068 | |
|  |  | Washout | | 136 | | 17 | | 38 | | 1.764 | | 1.024, 3.039 | |
| Stratification for gender** | |  | |  | |  | |  | |  | |  | |
| Females | | Unexposed | | 902 | | 918 | | 3,505 | |  | |  | |
|  | | Exposed | | 116 | | 60 | | 240 | | 1.328* | | 0.848, 2.077* | |
| Males | | Unexposed | | 1,048 | | 1,104 | | 4,087 | |  | |  | |
|  | | Exposed | | 81 | | 49 | | 127 | | 2.869* | | 1.699, 4.844* | |
| **Polypharmacy cohort** | |  | |  | |  | |  | |  | |  | |
| BP gap length 180days | | Unexposed | | 2,992 | | 3,095 | | 12,648 | |  | |  | |
|  |  | Exposed | | 336 | | 187 | | 664 | | 1.563 | | 1.197, 2.043 | |
| AKI: ICD-10 N17 | | Unexposed | | 2,926 | | 3,015 | | 12,451 | |  | |  | |
|  | | Exposed | | 331 | | 183 | | 648 | | 1.558 | | 1.19, 2.04 | |
| No fracture history | | Unexposed | | 2,885 | | 3,036 | | 12,244 | |  | |  | |
|  | | Exposed | | 229 | | 126 | | 459 | | 1.534 | | 1.112, 2.116 | |
| No 90days washout included for exposed period | | Unexposed | | 2,992 | | 3,126 | | 12,719 | |  | |  | |
|  |  | Exposed | | 336 | | 156 | | 592 | | 1.29 | | 0.978, 1.702 | |
| No 90days washout included for exposed period but added separately after each exposed period* | | Unexposed | | 2,992 | | 3,095 | | 12,653 | |  | |  | |
|  |  | Exposed | | 336 | | 156 | | 592 | | 1.459 | | 1.095, 1.944 | |
|  |  | Washout | | 240 | | 31 | | 66 | | 2.099 | | 1.396, 3.156 | |
| Stratification for gender** | |  | |  | |  | |  | |  | |  | |
| Females | | Unexposed | | 1,479 | | 1,496 | | 6,244 | |  | |  | |
|  | | Exposed | | 241 | | 125 | | 495 | | 1.509* | | 1.107, 2.055* | |
| Males | | Unexposed | | 1,513 | | 1,599 | | 6,408 | |  | |  | |
|  | | Exposed | | 95 | | 62 | | 164 | | 3.707* | | 2.315, 5.937* | |

IRR = Incidence rate ratio, 95%CI = 95% Confidence Interval, Length of follow-up (FU) is provided in person-years

* As described in the methods section of the manuscript, a 90-day washout period was added to the end of each continuous treatment episode to account for non-compliance and stockpiling. In this sensitivity analysis, this “washout” period was not included in the exposed patient time but treated as a separate post-exposure time window.

**This was a post hoc analysis following a reviewer comment. Due to a lack of sample size, 5-year age bands had to be used for age adjustment for the stratified analyses. Therefore, confounding by age may not be fully captured and IRR and 95%CI might be biased upwards.

Table S4a: Results from SCCS analyses for GI ulcer (hospitalisation cohort)

| **Analysis** | **Period** | **N(patients)** | **N(events)** | **Length of FU** | **IRR** | **95%CI** |
| --- | --- | --- | --- | --- | --- | --- |
| **Main analyses** |  |  |  |  |  |  |
| Unadjusted model | Unexposed | 1,019 | 1,085 | 4,553 |  |  |
|  | Exposed | 128 | 56 | 230 | 1.366 | 0.925, 2.017 |
| Age-adjusted model | Unexposed | 1,019 | 1,085 | 4,553 |  |  |
|  | Exposed | 128 | 56 | 230 | 1.269 | 0.849, 1.895 |
| **Sensitivity analyses** | |  |  |  |  |  |
| First event only | Unexposed | 1,019 | 967 | 4,553 |  |  |
|  | Exposed | 128 | 52 | 230 | 1.358 | 0.887, 2.08 |
| Pre-exposure washout period | Unexposed | 1,017 | 1,082 | 4,540 |  |  |
|  | Pre-exposure | 128 | <5 | 14 | 0.912 | 0.281, 2.955 |
|  | Exposed | 128 | 56 | 230 | 1.269 | 0.846, 1.904 |
| Risk windows | Unexposed | 1,019 | 1,085 | 4,553 |  |  |
|  | Risk window:  0-365 days | 128 | 30 | 122 | 1.203 | 0.765, 1.89 |
|  | Risk window:  366-730 days | 74 | 10 | 53 | 1.015 | 0.491, 2.098 |
|  | Risk window:  731+ days | 35 | 16 | 55 | 2.305 | 1.086, 4.893 |

IRR = Incidence rate ratio, 95%CI = 95% Confidence Interval, Length of follow-up (FU) is provided in person-years

Table S4b: Results from SCCS analyses for GI ulcer (polypharmacy cohort)

| **Analysis** | **Period** | **N(patients)** | **N(events)** | **Length of FU** | **IRR** | **95%CI** |
| --- | --- | --- | --- | --- | --- | --- |
| **Main analyses** |  |  |  |  |  |  |
| Unadjusted model | Unexposed | 1,453 | 1,580 | 6,914 |  |  |
|  | Exposed | 194 | 79 | 385 | 1.179 | 0.849, 1.637 |
| Age-adjusted model | Unexposed | 1,453 | 1,580 | 6,914 |  |  |
|  | Exposed | 194 | 79 | 385 | 1.067 | 0.764, 1.491 |
| **Sensitivity analyses** | |  |  |  |  |  |
| First event only | Unexposed | 1,453 | 1,377 | 6,914 |  |  |
|  | Exposed | 194 | 76 | 385 | 1.174 | 0.829, 1.663 |
| Pre-exposure washout period | Unexposed | 1,451 | 1,573 | 6,893 |  |  |
|  | Pre-exposure | 194 | 7 | 22 | 1.485 | 0.681, 3.239 |
|  | Exposed | 194 | 79 | 383 | 1.105 | 0.788, 1.55 |
| Risk windows | Unexposed | 1,453 | 1,580 | 6,914 |  |  |
|  | Risk window:  0-365 days | 194 | 38 | 201 | 0.945 | 0.641, 1.394 |
|  | Risk window:  366-730 days | 123 | 19 | 88 | 1.177 | 0.679, 2.037 |
|  | Risk window:  731+ days | 55 | 22 | 95 | 1.677 | 0.89, 3.157 |

IRR = Incidence rate ratio, 95%CI = 95% Confidence Interval, Length of follow-up (FU) is provided in person-years

Table S4c: Additional sensitivity analyses for GI ulcer (all cohorts)

| **Analysis** | **Period** | **N(patients)** | **N(events)** | **Length of FU** | **IRR** | **95%CI** |
| --- | --- | --- | --- | --- | --- | --- |
| **Frailty cohort** |  |  |  |  |  |  |
| BP gap length 180days | Unexposed | 1,403 | 1,535 | 6,366 |  |  |
|  | Exposed | 152 | 68 | 279 | 1.255 | 0.873, 1.805 |
| Serious GI ulcer (HES) | Unexposed | 1,006 | 1,066 | 4,523 |  |  |
|  | Exposed | 110 | 49 | 216 | 1.225 | 0.786, 1.908 |
| No 90days washout included for exposed period | Unexposed | 1,403 | 1,550 | 6,400 |  |  |
|  | Exposed | 152 | 53 | 245 | 1.013 | 0.683, 1.502 |
| No 90days washout included for exposed period, but added separately after each exposed period | Unexposed | 1,403 | 1,536 | 6,368 |  |  |
|  | Exposed | 152 | 53 | 245 | 1.084 | 0.726, 1.619 |
|  | Washout | 116 | 14 | 32 | 1.883 | 1.048, 3.383 |
| **Hospitalisation cohort** |  |  |  |  |  |  |
| BP gap length 180days | Unexposed | 1,019 | 1,084 | 4,552 |  |  |
|  | Exposed | 128 | 57 | 231 | 1.309 | 0.877, 1.954 |
| Serious GI ulcer (HES) | Unexposed | 717 | 739 | 3,174 |  |  |
|  | Exposed | 92 | 37 | 167 | 1.057 | 0.643, 1.74 |
| No 90days washout included for exposed period | Unexposed | 1,019 | 1,097 | 4,581 |  |  |
|  | Exposed | 128 | 44 | 202 | 1.021 | 0.66, 1.579 |
| No 90days washout included for exposed period, but added separately after each exposed period | Unexposed | 1,019 | 1,085 | 4,553 |  |  |
|  | Exposed | 128 | 44 | 202 | 1.101 | 0.707, 1.715 |
|  | Washout | 100 | 12 | 28 | 1.954 | 1.039, 3.675 |
| **Polypharmacy cohort** |  |  |  |  |  |  |
| BP gap length 180days | Unexposed | 1,453 | 1,578 | 6,911 |  |  |
|  | Exposed | 194 | 81 | 388 | 1.11 | 0.795, 1.55 |
| Serious GI ulcer (HES) | Unexposed | 1,059 | 1,120 | 4,973 |  |  |
|  | Exposed | 146 | 60 | 299 | 1.089 | 0.732, 1.621 |
| No 90days washout included for exposed period | Unexposed | 1,453 | 1,598 | 6,962 |  |  |
|  | Exposed | 194 | 61 | 337 | 0.838 | 0.583, 1.204 |
| No 90days washout included for exposed period, but added separately after each exposed period | Unexposed | 1,453 | 1,580 | 6,914 |  |  |
|  | Exposed | 194 | 61 | 337 | 0.903 | 0.624, 1.306 |
|  | Washout | 152 | 18 | 47 | 1.794 | 1.071, 3.006 |

IRR = Incidence rate ratio, 95%CI = 95% Confidence Interval, Length of follow-up (FU) is provided in person-years
